# Heterogeneous Integration Technology Drives the Evolution of Co-Packaged Optics

**DOI:** 10.3390/mi16091037

**Published:** 2025-09-10

**Authors:** Han Gao, Wanyi Yan, Dan Zhang, Daquan Yu

**Affiliations:** School of Electronic Science and Engineering, Xiamen University, Xiamen 361005, China; gaohan131@stu.xmu.edu.cn (H.G.); yanwanyi@stu.xmu.edu.cn (W.Y.); zhangdan@xmu.edu.cn (D.Z.)

**Keywords:** co-packaged optics, fan-out wafer level packaging, TSV, TGV, ion-exchange glass waveguide, femtosecond laser direct writing waveguides, optical interconnect, artificial neuromorphic, heterogeneous integration

## Abstract

The rapid growth of artificial intelligence (AI), data centers, and high-performance computing (HPC) has increased the demand for large bandwidth, high energy efficiency, and high-density optical interconnects. Co-packaged optics (CPO) technology offers a promising solution by integrating photonic integrated circuits (PICs) directly within or close to electronic integrated circuit (EIC) packages. This paper explores the evolution of CPO performance from various perspectives, including fan-out wafer level packaging (FOWLP), through-silicon via (TSV)-based packaging, through-glass via (TGV)-based packaging, femtosecond laser direct writing waveguides, ion-exchange glass waveguides, and optical coupling. Micro ring resonators (MRRs) are a high-density integration solution due to their compact size, excellent energy efficiency, and compatibility with CMOS processes. However, traditional thermal tuning methods face limitations such as high static power consumption and severe thermal crosstalk. To address these issues, non-volatile neuromorphic photonics has made breakthroughs using phase-change materials (PCMs). By combining the integrated storage and computing capabilities of photonic memory with the efficient optoelectronic interconnects of CPO, this deep integration is expected to work synergistically to overcome material, integration, and architectural challenges, driving the development of a new generation of computing hardware with high energy efficiency, low latency, and large bandwidth.

## 1. Introduction

Driven by the expansion of artificial intelligence computing clusters, data centers, and the future evolution of 6G networks, the global digital transformation process continues to deepen, with the demand for higher communication bandwidth and lower data transmission latency growing exponentially. In the early stages of its development, aluminum was primarily utilized as the interconnect material. However, as the feature sizes of integrated circuits continued to decrease, the limitations of aluminum interconnects became increasingly evident. In 1997, IBM developed copper interconnect technology. It has been demonstrated that copper offers approximately 40% lower resistance than aluminum and 100 times greater reliability. For this reason, copper is the primary material for both on-chip and board-level interconnects in integrated circuits [1]. The advent of large-scale integrated circuits has led to a proliferation of chip pins and a concomitant increase in the density of interconnects on PCB. The spacing and width of these interconnections have been measured to sub-millimeter and micrometer levels. Consequently, wire resistance, parasitic capacitance, and parasitic inductance increase lead to higher losses and delays. Copper interconnects demonstrate insertion loss levels as high as 5 dB/mm under 50 GHz signals [2]. When lane data rates exceed 200 Gbps, the copper trace loss on the PCB surpasses 6 dB. Conventional pluggable solutions incur over 20 dB of loss due to various transitions and path lengths [3]. A comparison of bandwidth loss and energy consumption from data transmission within chips, between chips and circuit boards, and between circuit boards reveals that optical fiber transmission in board-level communication reduces by four orders of magnitude compared to electrical transmission within chips and by two orders of magnitude compared to electrical transmission on the board [4]. Traditional electronic circuit technology based on metal interconnects is limited by bottlenecks such as significant signal delay, transmission loss, and electromagnetic interference, and it is difficult to meet the development requirements of modern high-performance electronic systems. Optoelectronic integration is mainly divided into two categories: integral and heterogeneous. Integral integration is mainly achieved by integrating diverse optical or electrical components into a common substrate [5]. Heterogeneous integration employs multiple process steps to machine different material systems into a substrate or to integrate different platforms [6]. As an important component of heterogeneous packaging, 2.5D/3D advanced packaging technology has great potential in improving interconnection density and reducing data latency, and has attracted the research interest of scholars [7]. CPO integrates optical components into electrical packages through advanced packaging technologies such as 2.5D/3D, which improve bandwidth density and power efficiency in data centers, especially as AI and machine learning (ML) applications increase the demand for complex computing and processing large data sets [8]. CPO builds an electro-optical collaborative transmission architecture by integrating the optical engine (OE) with the graphics processing unit (GPU), high-bandwidth memory (HBM), and application-specific integrated circuits (ASICs) inside the package. It uses millimeter-scale ultra-short electrical interconnects within the package to connect the core units directly. This direct connection significantly reduces both signal processing energy consumption and retiming requirements. At the same time, low-loss optical fiber replaces traditional board-level wires completely, which enables high-bandwidth transmission and low latency through direct optical fiber connection technology at the panel end. This structural innovation brings about a leap in system-level energy efficiency, ultimately. In optical Ethernet switch applications, the overall interconnection power consumption is reduced, providing a disruptive high-bandwidth, low-power transmission solution for data centers [9].

CPO technology realized through 2.5D/3D advanced packaging allows for shortening the interconnect length significantly, breaking through the limitations of planar integration, maximizing the integration density, and effectively suppressing parasitic effects, thus becoming the most promising technological pathway to achieve the next generation of high-efficiency and high bandwidth density. 2.5D/3D advanced packaging development enables the realization of inter-chip vertical interconnect stacking through TSV technology [10,11]. However, traditional intercalation is not favorable for high-frequency applications due to the high dielectric constant and loss tangent, which result in large losses. The high cost of the damascene process and the TSV through-hole preparation further limit the CPO technology [12]. Glass is considered a next-generation substrate material due to its lower dielectric constant and loss tangent, which can significantly reduce high-frequency signal loss [13,14]. Compared with silicon, glass allows optimization of the internal components, which affects thermal expansion coefficients and mechanical strength [15]. The simple process of manufacturing large-scale glass substrates contributes to endorsing glass substrates and TGV as the key technologies for 2.5D/3D packaging. The advantage includes excellent scalability for compactly integrating multiple optical and electronic chips into a single package. Similar to TSV technology, FOWLP allows EIC or PIC chips to be embedded in their adapter boards, enabling high-density interconnections and ultra-short signal paths in critical areas. In addition, FOWLP provides efficient heat dissipation and high compatibility with existing semiconductor manufacturing systems [16,17].

Optical waveguides play a vital role in PICs, and are utilized as basic optical components typically [18,19,20,21]. In recent years, femtosecond laser direct writing (FLDW) technology has shown significant promise for 3D fabrication with photonic devices on a variety of transparent substrates, including glass and crystals. FLDW waveguides are invisible to the naked eye, practically, because of their moderate refractive index variation (typically less than 10^−2^) and gradient refractive index distribution [22,23]. This technique provides a completely non-contact processing strategy inside transparent materials without additional processing steps, and allows the flexibility to change refractive index spatial distributions, resulting in the fabrication of devices with three-dimensional and complex structures [24,25,26,27]. However, it is not possible for this technique to avoid the disadvantages, including the high cost and low fabrication efficiency attributed to the complexity of the steps in the conventional lithography process. Planar optical waveguides fabricated on glass substrates using ion-exchange technology have significant advantages such as optical compatibility with glass fibers, suitability for mass production, and cost-effectiveness [28,29,30]. Glass waveguides provide the advantages of relatively simple processing, low transmission loss, a wide range of refractive indices, and the ability to be easily matched to glass fibers. Exhibiting excellent mechanical, thermal, electrical, and optical properties, glass waveguides have solved many difficult problems. For the future large-scale integration of CPO to form data centers, low-loss glass, inexpensive, and reliable glass waveguide fabrication techniques are becoming critical.

Optical technology forms the cornerstone of modern high-bandwidth communications, supporting the exploding demand for data exchange, especially in data centers and AI computing clusters. As the key platform for realizing next-generation CPO architectures, silicon photonics (SiPh) is expected to provide low-cost, high-performance, integrated optical transceivers and processing cores for data communications. The SiPh platform is capable of integrating various functional components [31,32,33,34,35,36]. However, the large-scale application of SiPh in CPOs faces a fundamental challenge with low-loss and high-efficiency coupling to optical fibers. This is mainly attributed to the significant mode size mismatch between standard optical fibers and submicron silicon waveguides. The OE and the electronics chip are packaged tightly, and the coupling efficiency directly determines the system’s power budget, transmission distance, and overall energy efficiency (pJ/bit). It is the critical factor in determining whether it can replace traditional pluggable optical modules and meet higher bandwidth requirements in the future. Fiber-to-chip interfaces depend mainly on grating couplers or edge couplers [32,37]. Although grating couplers require less package alignment, it is difficult for them to meet the requirements of CPO for high performance, low power consumption, and mass production due to their low coupling efficiency, narrow wavelength bandwidth, high polarization sensitivity, and complex manufacturing process [38,39,40]. In contrast, edge couplers are considered to provide a considerably more promising solution for realizing the high-density, low-loss optical I/O required for CPO because of their potentially high efficiency, wide wavelength, polarization insensitivity, and relatively simplified process. For CPO applications, researchers have developed a variety of high-efficiency edge coupler structures [41,42,43]. It is the core goal of these technologies to achieve near-lossless optical mode conversion, providing the basis for high-throughput, low-latency, and energy-efficient optical interconnects for a data-driven future.

Advances in glass substrate and waveguide technologies have overcome key barriers to CPO in high-frequency and high-density integration schemes. However, the energy efficiency challenges of the computing units themselves still require revolutionary breakthroughs. Brain-like intelligent computing is inspired by human brain functions and aims to build non-biological systems with brain-like functions. The development of ultra-low-power computers with autonomous learning and cognitive capabilities will lead to future technological advances [44,45,46]. Reconfigurable photonic devices are an essential part of next-generation optical technologies and have important applications in integrated photonics, nanophotonics, metasurfaces, and neuromorphic photonics [47,48,49,50,51]. As a major trend in AI and ML, photonic computing has received significant attention for energy efficiency in data transfer, but photoconversion and repetitive data accesses slow down the overall performance and cause additional energy loss [52,53,54,55]. Historically, memory devices have struggled with nonvolatility, but photonic memories based on phase-change materials (PCMs) can effectively address both compatibility and nonvolatility issues. In this way, complex neuromorphic systems can be modeled to support versatile and diverse artificial neural networks and provide an avenue for the development of AI computing clusters [56,57,58].

**Figure 1:**
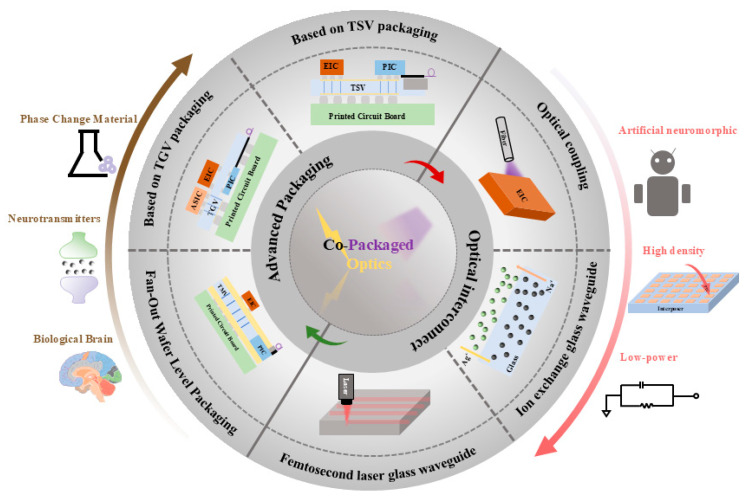
Heterogeneity Drives CPO Technology Evolution.

In this paper, we review the recent progress of CPO technology, investigate the performance differences brought by different packaging and glass waveguide technologies, and explore the role of coupling methods in promoting the development of CPO technology, as shown in Figure 1. With the future development of CPO technology, the innovative exploration of the synergistic heterogeneous integration of “optical-electrical-computing”, based on the integrated storage and computing mechanism of artificial neuromorphic photonic memory and the low-loss optical interconnect characteristic of CPO, will help overcome the obstacles faced by the future computing architecture of data centers and build a scalable hyperscale data-center energy efficiency optimization paradigm that provides a possible path.

## 2. Advanced Packaging Technology

**Figure 2:**
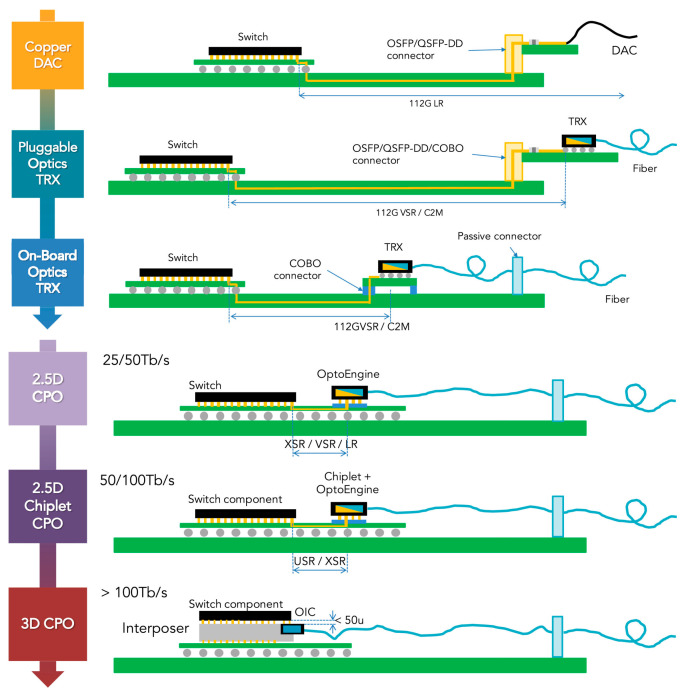
CPO packaging technology roadmap [59].

Nowadays, mature optical interconnect solutions include pluggable optical modules and on-board optical modules, but their integration density and data capacity are relatively low, and their power consumption is relatively high. The new generation of high-speed, high-density, and large-bandwidth optical interconnect technology is the key to supporting massive data communication in future switches, servers, and data centers. Chiplet technology has become a reliable technological solution to address these issues. As shown in Figure 2, with the development of CPO technology, the chip has been enabled to significantly increase the integration density and shorten the length of interconnect lines between chips [59]. With the advantages of low power consumption, low cost, and high capacity, it has become the mainstream development direction of optical interconnect [60,61]. With the rapid increase in bandwidth and energy-efficiency requirements in data centers and high-performance computing, CPO technology has become an essential development direction for next-generation optical interconnect solutions owing to its ability to significantly shorten electrical interconnect distances, reduce power consumption, and increase bandwidth density.

However, the successful implementation of CPO is highly dependent on breakthrough advanced packaging technologies. High-density heterogeneous integration of OE, complex thermal management requirements, and high-speed signal integrity assurance, as well as overall reliability and cost control, are the core challenges for CPO. Advanced packaging solutions that enable high-density interconnections, excellent thermal dissipation, and heterogeneous integration are key enabling technologies to address these challenges. FOWLP, 2.5D/3D integration technologies based on TSV, and new packaging platforms based on TGV have become a hot topic in research, development, and industrialization due to their unique technological advantages and their great potential to achieve high-performance, high-reliability, and low-cost CPO integration solutions.

### 2.1. Based on FOWLP Technology

FOWLP is an advanced packaging technology with excellent scalability for integrating different photonic and electronic components. EIC and PIC provide direct embedding into FOWLP interpolators for heterogeneous integration modes with ultrashort signal paths. FWOLP enables efficient heat dissipation and is compatible with current semiconductor fabrication processes [16]. Innovative explorations in silicon photonics play an important role in FOWLP technology. Silicon photonics allows the production of photonic components with low cost and high accuracy by utilizing the mature CMOS fabrication infrastructure. In addition, FOWLP technology enables dense packaging [62,63]. Heterogeneous integration of FOWLP between EIC and PIC has a positive effect on cost reduction and efficiency improvement [64]. The heterogeneous integration platform allows for improved overall signal performance by eliminating the need for discrete components and preventing damage to electrical signals. Tight integration of EIC and PIC enables high-speed interconnections with only 0.05 dB of loss at high frequencies up to 28 GHz. The EIC interconnects to the bottom substrate with less than 0.5 dB of loss as the frequency scales up to 56 GHz. The platform supports high signals above 100 Gbps. The platform supports high-speed signals over 100 Gbps and can be further integrated with larger ASICs to enable larger data centers and higher performance.

The rational design of the organic substrate can further optimize the platform performance [65]. On top of the 2.5D organic substrate, a micro-ring modulator (MRM), photodetector (PD), horizontal coupler, and fiber array (FA) are integrated for high-speed interconnections. The designed 12 × 12 mm^2^, 850 µm organic substrate contains eight high-speed signal channels with a 3 dB bandwidth of more than 30 GHz on a 10-layer substrate. The high-quality substrate was fabricated with no visible texture or form factor defects, and high-speed signals can be realized in the substrate with 56 Gbps NRZ OOK transmission with a signal-to-noise ratio (SNR) of about four, proving that the organic substrate has good CPO optical transceiver module capabilities. Continued growth in data traffic means continued development of high-speed channels for ASICs. The 1.6 Tbps Silicon Photonic Integrated Circuit (SIPIC) with 16 optical channels (1310 nm) and single-channel 106 Gbps PAM4 performance achieves a smaller footprint and allows for higher density integration in the same area to achieve better performance [66]. Thirty-two hybrid distributed feedback lasers (DFBs) were fabricated using a hybrid silicon laser platform, achieving 20 mW output power at 80 °C with less than a 5% increase in bias current and a 20-year lifetime. The integrated V-shaped array achieves smooth sidewalls through the use of passive SMF28 alignment, which minimizes coupling losses.

**Figure 3:**
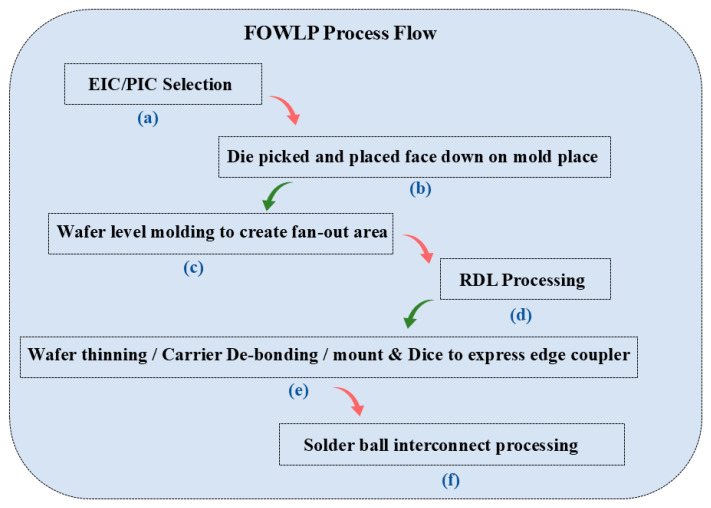
FOWLP process flow for OE packaging.

TSV technology is typically used to enable package-level integration, which allows for high manufacturing costs and consumes additional PIC area. In contrast to the high manufacturing costs associated with TSV, FOWLP solutions that integrate PIC with edge couplers for the first time can achieve up to 50% cost reductions to meet the needs of an optical engine, which is significantly different from other solutions [67]. Edge coupling is optimized for high-performance and high-integration packages. While edge coupling offers better performance than vertical coupling, contamination protection of the coupling structure during the packaging process is a significant problem. For this reason, we have innovatively designed a cantilever structure in the PIC coupler region. Compared to vertical grating couplers or edge couplers without cantilever structures, the performance of suspended couplers is excellent [68]. The structure acts as a physical barrier during the molding process, effectively preventing molding compounds from penetrating and contaminating the precise coupling surface. Upon completion of packaging, the cushioning structure is removed, and the coupler is exposed through a precise deep groove cutting process. Experiments have demonstrated that non-destructive exposure can be achieved at different groove widths (50 μm, 80 μm, 100 μm), which ensures the cleanliness and optical performance of the coupling interface. The entire package structure has been tested and proven to have excellent mechanical reliability with no cracks or delamination. For electrical interconnections, the design is optimized for the high-speed data transmission required for system-in-package (SiP). Structures such as the GSSG coplanar waveguide (CPW) ensure the integrity of high-speed signal transmission. The manufacturing process shown in Figure 3 employs a grain-first FOWLP process route and was implemented on a 300 mm state-of-the-art production line, demonstrating the solution’s manufacturability and mass-production potential.

While TSV technology has a place in package integration, its inherent high cost and encroachment on the PIC area are becoming increasingly prominent in the quest for high-density, low-cost optical engine packaging. In this context, exploring alternatives to TSVs is not only a matter of optimizing cost but also of finding a technology path that enables the integration of edge couplers. The value of the FOWLP solution lies not in simply lowering cost in fundamentally solving the core contradiction of enabling high-performance, contamination-free edge coupling in embedded packages through unique structural innovations and tooling-advancement processes. Instead of simply reducing costs, the FOWLP solution fundamentally solves the core contradiction of realizing high-performance, pollution-free edge coupling in embedded packages through unique structural innovations and mold-first processes. In addition, FOWLP not only circumvents the pain points of TSVs but also provides the possibility of realizing high-efficiency CPO in compact spaces, which lays the groundwork for subsequent advantages in high-performance interconnections and system-level integrations.

**Figure 4:**
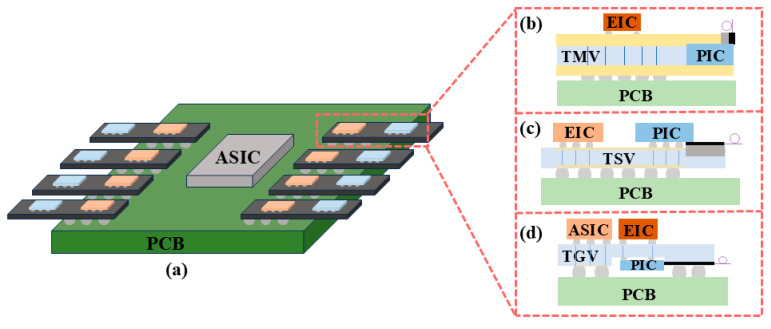
(**a**) Switch composed of 2.5D advanced packaging; (**b**) TMV-based, (**c**) TSV-based, and (**d**) TGV-based advanced packaging architectures.

As shown in Figure 4a,b, the FOWLP-based OE platform supports wireless bonding of EIC and PIC integrations to minimize signal loss and improve signal integrity [69]. In the PIC embedding platform, the EIC is encapsulated on the top surface, and a front redistribution layer (FRDL) and a backside redistribution layer (BRDL) are designed for high-density interconnections. The EIC is packaged directly on top of the PIC through an Under Bump Metallurgy (UBM) on the FOWLP FRDL to achieve the smallest path. RF losses are minimized during the interconnect between the high-speed driver and the Transimpedance Amplifier (TIA). Through-mold vias (TMVs) are available for interconnections between the bottom and top of the OE package to achieve rates of 224 Gbps/lane. The photonic engine contains eight high-speed channels with a receive capability of 1.79 Tbps and it has achieved good data-transfer performance in 112 Gbaud NRZ and 224 Gbaud PAM4 tests. By expanding the number of channels in the PIC or increasing the number of PICs in the FOWLP, the total data transfer rate of the OE could be further increased, which will help to reduce the latency of future AI/ML optical interconnects.

Recently, the world’s first 51.2 Tb/s co-packaged optical (CPO) switch prototype was introduced, enabling a paradigm shift in data-center optical interconnects through heterogeneous 3D integration of silicon photonic chips [9]. The solution employs a double-sided FOWLP that integrates eight CMOS silicon photonic engines (O-band channels per engine) with a 512-channel switch chip based on copper pillar bump interconnects and through-mold vias (TMVs). By replacing the traditional TSV technology, it reduces the electrical interconnect distance to the sub-millimeter level while avoiding high process complexity and significantly reducing signal attenuation. Combining the thermally separated construction of an external laser module (RLM) with a blind-mate fiber connector design, all 64 channels were activated. The energy-efficiency ratio of 0.9 pJ/bit reduces power consumption by 50% and increases bandwidth by 100% compared to plug-and-play solutions. Its polyimide dielectric layer warpage control technology is compatible with standard semiconductor packaging processes, providing a key technology pathway for industrializing CPO technology in hyperscale data centers and laying the groundwork for a single-channel 200 Gb/s evolution.

The intermediary layer plays a pivotal role as a crucial intermediate substrate, facilitating seamless electrical connectivity and signal transmission by connecting multiple chips. Among the various intermediary layer technologies, organic intermediary layers emerge as a cost-effective solution, offering scalability to support larger chip sizes and high input/output (I/O) densities [70,71]. Fan-out mediator layer substrate technology, based on 3D printing technology, enables high-density heterogeneous integration through the innovative use of curved through-hole designs [72]. The fabrication of organic substrates with embedded curved through-holes can be accomplished directly through the use of projected microstereoscopic light-curing 3D printers. This method overcomes the limitations of the traditional RDL lamination process, which involves multiple fabrication steps. A key benefit of this approach is the ability to establish vertical interconnections between the chip and the substrate in a single step. The integration of curved through-holes facilitates direct connection between the chip and the package substrate, thereby eliminating the need for multi-layer RDL. Concurrently, the groove-patterned pad facilitates simultaneous metallization through electroplating, integrating chemical copper plating with mechanical polishing to create a low-resistance pathway. Furthermore, the device under consideration facilitates high-density fan-out wiring, thereby reducing the through-hole resistance to a minimum of 0.81 Ω. This value is a substantial reduction of 62% in comparison to that of 90° straight through-holes. Additionally, the insertion loss at 40 GHz is recorded to be less than 0.5 dB. This interposer is compatible with standard copper pillar bump bonding. A 15-micrometer copper pillar, in conjunction with a 20-micrometer Sn/Ag solder cap, has been demonstrated to facilitate reliable interconnections at 280 degrees Celsius, exhibiting a series resistance of 1.85 ohms. This technological advancement significantly reduces the time required for traditional prototype development, from several weeks to a single day. Consequently, it offers a rapid and cost-effective fan-out solution for multi-chip heterogeneous integration in CPO systems; the progress is summarized in Table 1 below.

The industry’s first 3.2 Tbs optical engine with on-chip integrated MUX-DMUX for high-bandwidth switching network systems represents a significant advancement in the field [73]. The implementation of a state-of-the-art 3D packaging fan-out technology has enabled the successful integration of four 800 G EICs and a single 3.2 T PIC, facilitating the utilization of wafer-scale redistribution layers (RDLs) for the purpose of achieving a compact design and the establishment of low-parasitic electrical connections. Double-sided RDLs were utilized in the construction of 600 μm ultra-short-distance interconnects between chips, thereby reducing the insertion loss in the 26 GHz band from 1.84 dB to 1.2 dB in comparison to conventional gold wire bonding solutions. For the first time, a large-scale substrate integration solution that is repairable was created. This solution integrates eight optical engine modules on a 110 × 110 mm^2^ organic substrate. The integration of a customized metal frame and μLGA socket has been demonstrated to achieve 5 μm alignment accuracy, thereby effectively addressing the thermal–mechanical stress problem of large-scale packages during reflow soldering. This approach has been shown to result in a 40% reduction in warpage. The integration of a 24-channel fiber array unit (FAU) at the periphery of the PIC chip, facilitated by high-density fiber-coupling technology, has been demonstrated to result in a single-channel coupling loss of <1.5 dB. A top-mounted heat sink is also employed to optimize thermal management and optical alignment stability concurrently. This technology has been validated in a 25.6 T CPO switch system, achieving a 25% reduction in system-level power consumption at full 100 G PAM4 port traffic compared to traditional pluggable module architectures. This development signifies the transition of CPO technology from the initial proof-of-concept phase to its industrial implementation. This architecture provides a CPO solution for hyperscale data centers that is capable of mass production. The packaging and modular socket design of the 51.2 T switch system has been shown to improve manufacturing yield and field maintainability, thereby establishing the technical foundation for next-generation 51.2 T switch systems.

### 2.2. Based on TSV Packaging Technology

The increasing data traffic, driven by HPC, has been shown to result in higher-frequency losses in ASICs and optical subassemblies (OSAs) [74]. Existing optical interconnect solutions enable the assembly of optical socket modules with discrete transceiver components on PCB boards. Due to their limited integration density, modest data capacity, and substantial power consumption, these components are constrained in their electrical connection beyond the packaging of computing nodes, thereby hindering scalability to accommodate future data rate demands. This necessitates the development of a novel generation of high-speed, high-density, and high-bandwidth interconnect technologies to facilitate future server and data-center communications [75]. 2.5D CPO technology primarily utilizes silicon interposers or glass interposers as high-density interconnect platforms. It integrates EIC and PIC side by side on the interposer through flip-chip bonding. The integration of high-density RDLs and TSV within or on the surface of the interposer facilitates ultra-short distance, high-bandwidth electrical interconnection between EIC and PIC in the planar dimension, as illustrated in Figure 4c. The integrated interposer is then vertically connected to the underlying package substrate or PCB via a ball grid array (BGA) or other method. Heterogeneous integration facilitates enhanced precision in the alignment of electronic and photonic components, leading to a reduction in interconnect losses at elevated frequencies. Consequently, this integration fosters the augmentation of both the quantity and the velocity of high-speed channels in optical transceivers.

The development of TSV interposers has emerged as a pivotal area of research and innovation, owing to their transformative impact on performance enhancement. In comparison with conventional organic or ceramic packaging substrates, TSV interposers offer a substantial advantage in terms of their remarkably high wiring density and I/O bandwidth, representing a significant advancement in the field. This high interconnection density has increased the number of signals that can be transmitted per unit area, thereby providing fundamental support for meeting the stringent requirements of applications such as HPC, AI accelerators, GPUs, and HBM integration for massive data throughput [76,77]. The fundamental value of TSV technology lies in its ability to fundamentally reshape chip integration. The process entails the vertical penetration of the conductive channel of the silicon interposer, thereby facilitating “direct interconnection” between chips and significantly reducing the interconnection distance. This approach has been demonstrated to significantly reduce signal-transmission delay and power consumption, thereby enhancing the overall performance of the system. Moreover, it has been shown to unlock advanced heterogeneous integration capabilities, which is a key advantage in modern technological applications. Heterogeneous integration facilitates the seamless integration of bare chips that are manufactured on disparate types of semiconductor substrates, employing varying process nodes, and exhibiting diverse functionalities, into a cohesive package through the implementation of high-density interconnection of TSV interposers. This “beyond Moore’s Law” integration approach enables the system to flexibly combine the most optimized technology modules to achieve unprecedented levels of performance, power consumption, cost, and functional diversity.

Given the critical role of TSV interposers in achieving high-density heterogeneous integration, especially in 2.5D packaging architectures, robust and reliable structural and material designs for specific applications are crucial. The process of 2.5D packaging entails multifaceted challenges, including thermal management, mechanical stress, electrical signal integrity, and long-term reliability. Consequently, the design must comprehensively consider the material selection, structural design, thermal interface materials, bottom fill materials, and overall mechanical support solutions of the interposer at the system level to ensure sustained electrical performance and mechanical stability. A significant body of research has been dedicated to the verification and enhancement of the process maturity of 2.5D silicon interposer technology. These studies encompass the critical process steps involved in the overall procedure, encompassing TSV manufacturing, wafer thinning, chip/interposer bonding, micro-bump formation, and the connection between the interposer and the packaging substrate. The objective is to attain high yield and high consistency while adhering to stringent reliability standards [78,79,80].

The primary challenge in implementing silicon photonics technology in data-center interconnects pertains to the necessity of attaining effective collaboration between the photonic PIC and the EIC. Given that computation invariably commences and concludes within the electrical domain, photonic links necessitate the implementation of dedicated driver circuits to establish a high-performance interface with the electronic system. The degree of integration of driver electronics and photonics directly correlates with the bandwidth and energy efficiency of the transceiver. The primary constraints pertain to parasitic effects present within the receive path, the introduction of noise, and bandwidth limitations.

To overcome this challenge, the PIC and EIC are integrated nearby on a silicon-based interposer through a process known as flip-chip bonding. The interposer functions as a high-density interconnection platform, employing micron-level wiring to interconnect chips and connecting the top surface pads to the back package substrate TSV [81]. The interposer pad pitch can be precisely matched with the I/O pads of the PIC/EIC, supporting μbump or copper pillars to achieve low parasitic, high-density connections, while supporting multi-chip integration to expand system functions. A four-channel WDM receiver employs four demultiplexing microdisks, which are coupled with the bus waveguide at the PIC end. The drop port is connected to the germanium PD. The PD output is connected to a single-channel bare chip TIA via a copper pillar bump-interposer trace. The TIA output is routed to the back through the interposer TSV and connected to the PCB via the BGA. The high-speed signal is transmitted by the microstrip line to the SMA interface, supporting 11.3 Gbps transmission per channel. After the deduction of 7.4 dB of edge coupler and microdisk losses, BER < 1 × 10^−9^ was attained at 5 Gbps/−10.5 dBm received power. This finding substantiates the scalability of 2.5D integration and delineates a technical pathway for integrating silicon photonics into microprocessors.

The utilization of electrical interconnection methods is constrained by two primary factors: the presence of significant delay and severe attenuation at high frequencies. Consequently, optical interconnects are similarly constrained at high transmission rates. The employment of high-resistance silicon as an intermediate layer facilitates the fabrication of the thinnest wires and smallest vias through the silicon intermediate layer process. High-density μbumps/microspheres are utilized for interconnections between EICs and PICs to enhance reliability and expand transmission bandwidth [82]. Following the implementation of electrical performance optimization measures, the insertion loss of the entire link is maintained within −1.55 dB at 40 GHz, thereby ensuring the generation of a clear PAM 4 analog eye diagram at 32 Gbaud/64 Gbps. The transmitter has been demonstrated to attain a clear eye diagram at a PAM 4 signaling rate of 64 Gbps per channel while sustaining a power consumption of 6 pJ/bit. This development aligns with the broader initiative to promote higher-speed PAM4 signaling. The architecture can be applied to 1.6 T/3.2 T CPO optical engines to enhance the scalability of the architecture.

To promote higher-speed connections, a set of two grooves or U-shaped grooves was designed on the silicon interposer [83]. Because μbumps possess low capacitance and inductance, these bumps are designed between the two U-shaped grooves to allow electrical routing between the PIC and EIC while enabling high-frequency electrical connections. The silicon interposer provides facilitation connections with a bandwidth exceeding 100 billion bits per second. In the case of higher-density connections, the routing of electrical signals through the TSVs of the Si interposer is a viable option.

Broadcom’s single optical engine has been demonstrated to achieve 32-channel full-duplex transmission, with a rate of 100 Gb/s per channel and an aggregate bandwidth of 3.2 Tb/s [84]. The system’s energy efficiency is optimized to 5–10 pJ/bit, which is more than 50% lower than that of traditional pluggable modules. The electrical channel insertion loss between the ASIC and the optical engine is only 2–3 dB, which is 90% lower than that of modular solutions. Concerning optical performance, the transmitter TDECQ is less than 2 dB (which is superior to the IEEE 3.4 dB upper limit), and the extinction ratio attains 4 dB. The receiver has been found to meet the bit error rate standard at −10 dBm OMA and has been determined to reserve a 9 dB dynamic margin. The TSV finishing process is employed to attain a 130 μm pitch copper pillar interconnection, ensuring precise alignment with the CMOS/PIC bump density. Concurrently, a 127 μm pitch pluggable optical fiber connector is employed to integrate 72 fibers within a 14.43 mm coastline. A single switch encapsulates four optical engines, thereby achieving a total bandwidth of 12.8 Tb/s. When combined with the RLM architecture, which has four channels driven per laser, this provides a scalable foundation for the evolution of channel rates to 200 Gb/s.

3D CPO technology employs a three-dimensional stacking architecture to integrate EIC and PIC in a vertical direction. Flip-chip stacking is a well-known heterogeneous integration process in the industry that facilitates the interconnection of different chips in either a face-to-face or back-to-back configuration. This integration method has been shown to significantly shorten the interconnection distance between EIC and PIC, thereby effectively reducing the parasitic parameters introduced by the interconnection. This, in turn, has been demonstrated to improve the transmission performance of high-frequency/high-speed signals and to achieve higher system integration density. The crux of three-dimensional integration can be attributed to the design and fabrication of high-performance interposers. Passive interposers are frequently employed to facilitate chip-to-chip interconnection and fan out fine-pitch chip signal pins to a looser package-level pitch [85].

TSV is a pivotal technology for achieving vertical interconnection within the interposer. Active photonic interposers further integrate PIC functions on the basis of providing passive interconnection functions [86]. The vertical transmission and interconnection of electrical signals can be achieved through structures such as RDL, TSV, and microbumps or solder balls. TSV is regarded as the core enabling technology for three-dimensional integration, providing key support for the realization of highly miniaturized and complex next-generation systems. It is a promising solution for continuing the development path of “More than Moore.” Nevertheless, a primary challenge in the development of 3D CPO technology is the construction of reliable and high-performance three-dimensional circuits and systems. Three-dimensional integration has been demonstrated to alleviate the bottleneck of traditional interconnection by shortening the interconnection length and reducing gate delay. However, numerous technical difficulties, such as thermal management and stress control, must still be overcome during the implementation process.

The advent of high-speed data centers and optical interconnection technology has rendered silicon photonics 3D integration a pivotal solution for CPO. This integration enables the reduction of interconnection distances between electro-optical chips, thereby enhancing bandwidth and integration density. The TSV three-dimensional optoelectronic integrated packaging solution, based on a high-resistance SOI substrate, provides a key technical path for high-speed optical interconnection systems of 400 Gbps and above [87]. The study utilized high-resistance SOI wafers to prepare TSV structures with a diameter of 25 μm and a depth-to-width ratio of 4.2:1 by means of deep reactive ion etching. Additionally, the coplanar waveguide and microstrip line design was optimized to reduce RF loss. The experimental results indicate that the 3D packaged test vehicle, which was assembled by thermocompression bonding, exhibits an insertion loss (S21) of less than 3.5 dB in the 50 GHz frequency band and a return loss (S11) of more than −13 dB. The device has been demonstrated to support 50 Gbaud PAM4 modulation and meet the requirements of 400 Gbps optical modules. In comparison with conventional wire bonding methodologies, this innovative approach reduces electrical interconnect lengths to the 100 μm level, thereby significantly mitigating parasitic effects. Furthermore, the integration of wafer-level electroplated micro-bumps facilitates high-density integration, thereby providing a compact solution for applications such as optical transceivers and optically controlled phased array microwave systems. This work not only validates the high-frequency reliability of TSV in optoelectronic co-packaging but also highlights the key value of coordinated optimization of high-resistance silicon materials and advanced interconnect processes in overcoming the bottleneck of next-generation terabit/s bandwidth.

Conventional 2.5D integration grapples with limitations, including a constricted high-frequency response and downward-facing optical interfaces. However, 3D integration based on active photonic adapters has emerged as the prevailing approach, achieving vertical interconnection through TSV and RDL. Notably, this marks the inaugural instance in which edge couplers have been co-designed and manufactured in conjunction with TSVs on SOI substrates [88]. The adoption of an RDL-TSV-RDL configuration, combined with the optimization of the single-hole TSV layout and RDL length, has resulted in an insertion loss of less than 0.35 dB at 67 GHz. Excellent signal integrity was achieved at 112 Gbps eye diagrams. The development of TSV preceded the EC process, effectively preventing the risk of EC contamination in last-via technology. The adapter exhibited commendable high-frequency characteristics and optical performance, thereby establishing a research foundation for the large-scale implementation of system integration based on 3D CPO technology. The relevant progress is illustrated in Table 2.

The recent surge in demand for bandwidth and computing power required to support large AI models and HPC has led to significant advancements in heterogeneous integrated packaging, which has emerged as a pivotal approach to overcoming the limitations of Moore’s Law. However, the warpage problem caused by large-size packaging and high density has restricted assembly yield and reliability, and the power consumption has risen to over 2000 W, posing a severe challenge to thermal management [92,93,94]. In 2.5D advanced packaging for HPC and AI, TSMC’s CoWoS-R technology achieves high-density heterogeneous integration through an organic RDL interposer, supporting large-scale integration of multiple system on a chip (SoC) chips and HBM storage [95]. This platform reduces the RDL line width/spacing from 2/2 μm to 1.4/1.4 μm and decreases the through-hole size from 8 μm to 3 μm. Consequently, the line density is increased from 1100 lines/mm to >2200 lines/mm, thereby providing substantial support for high-speed UCIe (32 Gbps) and HBM3/4 (6–10 Gbps) interconnects. This miniaturization, however, comes with a trade-off between RC impedance and line length. In HBM, long-distance transmission with thin lines leads to an increase in RC impedance, thereby limiting high-speed signal integrity. Furthermore, eye diagram tests demonstrate that a 1.4 μm line width supports only 8 Gbps at a line length of 6.5 mm. Consequently, the line width selection must be optimized based on the specific application scenario.

The issue of warpage is particularly salient in the context of large-sized packages. A 1.3-fold increase in substrate size has been shown to result in a 1.3-fold increase in room-temperature warpage, while a 1.8-fold increase in CoW size has been demonstrated to cause a 2.2-fold increase in warpage [96]. In order to address the issue of warpage, TSMC has proposed a solution that involves the implementation of a warpage compensation layer structure, in conjunction with a sealing ring accessory. This approach has the objective of reducing the warpage of a 5.5-reticle (5.5×) package on a 110 × 110 mm^2^ substrate by 30–45%. Concurrently, the uniform stress distribution of the organic interposer and the 99.5% yield of CoW bonding establishes the foundation for large-scale integration.

Another significant challenge pertains to thermal management. For CoWoS-R packages with power consumption >1000 W, indium metal TIM (thermal conductivity > 80 W/mK) has been shown to achieve a junction temperature of 105 °C and a thermal resistance of 0.0108 °C/W at 1866 W power consumption. This result is superior to those of graphite film (>20 W/mK) and liquid metal (>8 W/mK) [97]. The application of impingement liquid cooling has been demonstrated to enhance heat dissipation capacity to 2112 W. However, the high voltage drop issue must be addressed to optimize its performance. The innovative direct silicon liquid cooling technology eradicates the thermal resistance of the TIM interface by etching a micro-pillar array on the back of the SoC and connecting the liquid cooling cover with an elastic sealant. The CoWoS-R platform demonstrated the capacity for effective heat dissipation, with IMC-Si achieving 3.4 kW of heat dissipation (power density of 2.5 W/mm^2^) at a flow rate of 10 LPM. Additionally, the helium leakage rate of the sealant was found to be lower than the critical value of 4.4 × 10^−6^ Pa·m^3^/s, a benchmark that was met after 2000 cycles (−40–125°C) and 1000 h. These findings are in alignment with the reliability requirements established for data centers [98].

In 2.5D heterogeneous integration for CPO, Intel’s EMIB-T technology reconstructs the power supply path through silicon via TSV bridge chips, thereby solving the power supply bottleneck of ultra-large form factor [99]. The conventional EMIB configuration exhibits a high voltage drop along its cantilever power supply path. In contrast, the EMIB-T design facilitates direct current flow from the bottom of the package through the TSV bridge to the HBM chip, thereby markedly enhancing power-supply efficiency. This technology supports a 45 μm interconnection pitch and has passed reliability verification, including pre-conditioning and thermal cycling 1500 times in a 77 × 56 mm package size. These findings lay the foundation for HBM4/4e integration and UCle-A 32 Gbps high-speed interconnection.

In response to the demand for sub-micron interconnection density, the multi-layer via architecture (MLV) innovatively divides the bonding layer vias into two categories [100]. The implementation of “power vias” that are directly connected to the power supply layer has been demonstrated to result in a reduction of losses. Additionally, the integration of “signal vias” that are connected to the bottom metal has been shown to facilitate hybrid bonding (HB) pitch reduction to 1 μm. In comparison with conventional stacked vias, MLV has been shown to reduce parasitic capacitance by 24% at a 3-μm pitch. Furthermore, MLV facilitates the penetration of the multi-layer dielectric stack through deep hole etching (>8 μm)-μm with a depth-to-width ratio of >20:1. This architecture is compatible with front/back power supply networks, thereby averting the degradation of the Q factor of RF passive devices caused by thinning of the top metal.

In the context of thermal management, low thermal resistance (LTR) wafer bonding technology has been shown to overcome the thermal limitations of conventional melt bonding methods [101]. Following a process of annealing at a temperature of 350 °C, the bonding energy of the newly developed bonding material is found to be a minimum of 400 mJ/m^2^, which is at least twice that of oxide bonding. Additionally, there is a 10-fold reduction in thermal resistance, with a value of 10^−3^ °C cm^2^/W, as compared to the SiO_2_ bonding layer, which has a thermal resistance of 1.8–2 × 10^−2^ °C cm^2^/W. Thermal simulation indicates that replacing conventional bonding materials in client products and AI accelerators can enhance heat dissipation capacity by 11.1% and 12.1%, respectively, thereby providing a pivotal solution for addressing hot-spot heat dissipation in 3D stacking. Intel employs three innovative technologies: the EMIB-T, MLV architecture, and LTR bonding to address the power supply efficiency, interconnection density, and heat dissipation bottleneck issues in ultra-large packaging. The combination of these technologies has been identified as a key factor contributing to Intel’s competitive advantage in the domain of 2.5D CPO packaging.

### 2.3. Based on TGV Packaging Technology

Glass interposers have been demonstrated to offer superior scalability for industrial applications in comparison with silicon interposers. This advantage can be attributed to their lower cost, higher integration density, and low dielectric constant. Furthermore, their excellent thermal and mechanical adaptability minimizes warping and cracking. Additionally, their excellent sealing and transparency make them a promising packaging material for next-generation semiconductor devices, demonstrating their significant potential in the future.

The SiPh platform, which integrates optical devices on Si chips and demonstrates high compatibility, has become the prevailing trend, as illustrated in Figure 4d. This type of packaging typically necessitates sealing and thermal control, reliability, and wavelength stability. It generally utilizes a package composed of ceramic materials. The substantial volume of the package, the high dielectric constant of the ceramic, the high thermal expansion coefficient, and the high power consumption are incompatible with the performance requirements of high-density integration. The utilization of surface photonic packaging with a photon binding-glass interposer (GIP) has been identified as a viable solution to this challenge [102]. The implementation of GIP on a printed circuit board utilizes the Ball Grid Array (BGA) technology. In order to achieve a highly reliable heat dissipation structure, the PIC and EIC are mounted on the BGA side of the GIP and sealed with a glass cover. The digital signal processor (DSP) is mounted on the front side of the general-purpose input/output (GPI) and connected to the electronic interface controller using high-speed electrical signals through TGV. The optical fiber, equipped with an FAU, is affixed to the periphery of the GIP. The PIC is linked to the fiber through a photonic wire and a glass waveguide, facilitating optical communication between the two components. The temperature of the PIC is managed by a high-density TGV that functions as a sealed HTB. This configuration facilitates the extraction of optical signals, electrical signals, and heat from a sealed space. The low thermal expansion coefficient and low dielectric constant are bound and sealed using photonic wires. Direct coupling of the PIC and glass waveguide has been demonstrated to achieve high efficiency in the C-band, with a range of −1.9 dB to −2.3 dB. The transmission line, in conjunction with the TGV, extracts high-speed electrical signals from the EIC, thereby providing 120 GHz high-frequency performance devoid of resonance. This effectively suppresses temperature variations between the EIC and PIC, thereby enabling thermal management and superior control of ultra-high frequency signals.

Glass exhibits a distinctive combination of properties that renders it particularly well-suited to address the challenges associated with co-packaging. Additionally, it possesses the capacity to incorporate optical waveguide interfaces, facilitating the pick-and-place assembly of PIC. Glass substrates with integrated planar IOX optical waveguides beneath the top surface of the glass provide well-defined optical interfaces in terms of position, allowing the assembled PIC to be directly evanescently coupled with low loss. Nevertheless, challenges persist in the precise control of cavity size and depth, as well as the concurrent fabrication of the substrate and the TGV. Consequently, the electro-optical substrate concept was proposed [89]. The system under consideration integrates optical interconnects, electrical RDL, and packaging TGV. Both high-volume ASICs and PICs will be connected to the RDL through electrical connections. The glass substrate is fabricated with different cavity depths, ranging from 30 μm to 140 μm. The photoresist is applied using a spray coating process to achieve uniform photoresist thickness on the cavity bottom and along the top surface. The thickness of the material is uniform at approximately 200 μm from the cavity wall. The optical interface is constituted by an evanescent wave coupler, which is situated between the silicon photonic chip and the glass waveguide that has been integrated into the substrate. Glass waveguides are configured for interconnection with optical fibers via fiber array connectors. The glass waveguides can be extended to the front panel, thereby replacing the fiber bridges. The employment of a photoresist spray coating process has yielded the successful creation of ultra-fine lines and spaces with a width of 5 μm within the glass cavity. This development paves the way for potential applications in 102.4 Tb/s data-center switches.

The 400 Gbps optical engine, based on the TGV interposer, has been demonstrated to overcome the high RF loss of the electrical interconnection between the printed circuit board and the optical engine. This innovation has also been shown to solve the problems of high-frequency signal loss and module maintainability through innovative design [90]. The core breakthrough of this technology is the simultaneous resolution of two significant challenges faced by the industry: high-frequency signal loss and module maintainability. The optical engine exhibits an S21 insertion loss of less than 1.5 dB at a bandwidth of 67 GHz, which is more than 5 dB better than that of traditional PCBs. The E/O bandwidth of the device reaches 51.8 GHz at 25 °C/85 °C. The optical engine module can be expeditiously replaced by means of the original 808 nm laser penetration welding technology. The solder joint melting time is less than 20 s at a power of 14.3 W. After replacement, TDECQ remains below 1.6 dB, and the 10 km transmission reception sensitivity of −8.3 dBm is maintained. These findings substantiate the hypothesis that the TGV glass substrate possesses inherent physical advantages in terms of suppressing RF loss and exhibiting favorable infrared transmission characteristics. The relevant progress is demonstrated in Table 2.

The advent of advanced manufacturing processes based on glass substrates has the potential to catalyze the development of higher speeds and bandwidths [103]. The integration of a cavity structure with a high aspect ratio TGV is achieved through the synergy of mechanical processing and wet etching. The cavity taper ratio can be controlled within the range of 0.1 to 0.6. The innovative use of spray photoresist combined with laser direct write exposure (LDI) technology overcomes the difficulties of traditional photolithography in deep cavities and realizes RDL of fine copper wiring at the bottom of the cavity. This technology offers a pivotal glass substrate solution for the low-loss, high-density optoelectronic co-packaging demanded by AI/HPC and data centers.

Glass substrates are poised to assume a pivotal role in facilitating high-density optoelectronic integration within the CPO framework. To facilitate the subsequent evolution of these technologies, it is imperative to surmount the impediments currently impeding the advancement of three-dimensional architecture and heterogeneous synergy. Current technologies facilitate the fabrication of integrated cavity-TGV and the implementation of deep-cavity micron-scale RDL routing. However, the stringent requirements of AI and HPC for computing power density and energy efficiency underscore the imperative for ongoing advancements in multi-layer heterogeneous stacking. This necessitates the development of cavity-in-cavity structures and three-dimensional TGV interconnect networks to facilitate the deep integration of optoelectronic chips, thereby overcoming the physical limitations inherent to planar layouts. In addition, it is imperative to optimize the thermal expansion coefficient matching of glass substrates to effectively mitigate photon–electron coupling losses. Moreover, the establishment of a comprehensive lifecycle reliability system is imperative to ascertain the stability of deep-cavity structures under dynamic loads through accelerated thermomechanical stress testing. Achieving these breakthroughs is imperative for the advancement of glass substrates from passive substrates to active optoelectronic fusion platforms, thereby facilitating the implementation of optoelectronic symbiosis architectures for data-center switches with a capacity exceeding 102.4 Tb/s.

The development of artificial intelligence has led to a gradual increase in the operating power level of data centers. The next-generation HPC platform, which targets extremely high speeds, has generated diverse power demands and requires an optimized power distribution network (PDN) to minimize losses and maintain power integrity [104]. The glass-substrate-embedded silicon deep trench capacitor (DTC) technology of the 3D stacked integrated voltage regulator (IVR) vertical power supply (VPD) has been demonstrated to yield ultra-high density and ultra-low parasitic parameters through innovative 3D integration [105]. The 3D architecture of the device under consideration embeds a 210 μm-thick, multi-terminal DTC chip within a 200 μm glass substrate. The fabrication of TGV and stacked microvias involves a process of laser ablation, utilizing a semi-additive method to create a vertical current path within the glass layer. Experimental verification has been undertaken to assess the performance of the solution, and the results demonstrate that it can support a total capacitance of hundreds of microfarads. This capacity is sufficient to meet the vertical power supply requirements of a 1 kW-class IVR. The system’s compatibility with the prospective augmentation of 3D stacking of 90 μm ultra-thin glass and 75 μm DTCs is also noteworthy. This model provides a solution for high-bandwidth, low-loss 3D power transmission in co-packaged optical systems.

Fujitsu Laboratories has achieved a significant milestone in the field of silicon photonic transceiver technology, with the development of the world’s first 16-channel × 25 Gb/s silicon photonic transceiver. This advancement has yielded a remarkable result, namely, 400 Gb/s aggregate bandwidth, which is a testament to the transceiver’s advanced capabilities. Furthermore, the transceiver exhibits an industry-leading bandwidth density of 363 Gb/s/cm^2^, a feat made possible by the implementation of a high-density bridging structure [91]. The 28-nanometer CMOS EIC and PIC are directly interconnected using flip-chip bonding, thereby reducing the electrical interconnection path to its shortest possible form. Concurrently, 100Ω differential signal lines are integrated through a glass ceramic interposer (GCIP) to suppress signal reflection and crosstalk. The optimization of power integrity (PI) and signal integrity (SI) facilitates the integration of the Tx/Rx independent power supply network with the on-chip capacitor layout, thereby reducing Tx-Tx and Rx–Rx crosstalk to 1.4 dB, and ensuring Tx-Rx crosstalk remains below 0.1 dB. The PIC integrates a 4 × 4 laser diode array (LD) and a silicon germanium detector, thereby realizing 32 channels of optical I/O through a grating coupler. The active alignment multi-fiber ferrule facilitates full functional integration within an area of 11 × 10 mm^2^. The 16-channel 25 Gb/s NRZ signal transmission was found to be BER < 10^−12^, yet the influence of the traditional MZ modulator suggests potential for enhancement of energy efficiency.

While the industrialization of 3D CPO technology has achieved breakthroughs, the path to next-generation systems with higher speeds and higher density still faces three core challenges. The solutions to these challenges will profoundly impact the direction of technological evolution. The prevailing energy efficiency challenges in conventional devices necessitate the investigation of innovative optoelectronic collaborative architectures to overcome the energy consumption per bit constraints at the system level. Moreover, 3D stacking has been shown to increase thermal density exponentially, thereby further complicating thermal stress management. As transmission rates and bandwidths continue to increase, the system’s heat flux density will gradually increase. The development of technologies aimed at enhancing the thermal conductivity of heterogeneous material interfaces, in conjunction with artificial intelligence (AI)-driven thermal stress models, is poised to assume increasing significance. These technologies are designed to facilitate pre-packaging predictions and prevent thermally induced package failures. As systems continue to evolve, high-frequency interconnects are approaching physical limits, and high-speed channels will face severe loss and crosstalk challenges. The advent of high-aspect-ratio TSV/TGV systems poses a significant challenge to the pursuit of continuous breakthroughs in ALD technology. Additionally, the degree to which industry ecosystem collaboration occurs will be a pivotal factor. The absence of co-packaging interface standards for engine and switch chips underscores the necessity for the establishment of an open, cross-enterprise interoperability framework. In order to effectively address the diagnostic challenges posed by 3D integrated systems, the utilization of wafer-level optoelectronic co-probe stations is imperative during the testing process. Achieving a generational leap from the current optical engine to the future 100 T-level system or beyond necessitates the comprehensive restructuring of the existing chain of “materials-design-manufacturing-verification.”

The vertical stacking of chips in three-dimensional packaging facilitates high-density integration and augments signal transmission performance. However, this approach concomitantly results in a substantial surge in power density, thereby rendering thermal management a pivotal challenge that hinders the reliability of high-capacity 3D CPO devices. Photonic components, including semiconductor optical amplifiers and micro-ring resonators, demonstrate a notable temperature dependence. A temperature variation of 20 °C is generally sufficient to cause a PIC to deviate from its operating range [106]. Conventional underfill materials utilized in packaging, such as epoxy resins, exhibit comparatively low thermal conductivity and are increasingly inadequate in meeting the demands of three-dimensional packaging. In order to address the aforementioned limitation, high-filler-content materials (typically exceeding 50%) with excellent thermal conductivity, such as aluminum nitride (AlN) [107] and boron nitride (BN) [108], can be incorporated into the epoxy resin matrix. In the context of 3D-stacked chips, the absence of direct thermal conduction pathways surrounding the un-bumped area at the periphery of the large-sized chip leads to a significant elevation in temperature within the package. Thermal bridge structures have been demonstrated to facilitate the connection between the un-bumped periphery of large-sized chips and the lid, thereby enhancing thermal management. Zhang et al. [109] addressed the thermal management challenges of 3D-stacked memory chips on processors based on this approach. The thermal bridge, which exhibited a square ring shape, was affixed to the un-bumped area of the processor via die-attach material, arranged in parallel with the memory stack. This connection was then facilitated through the thermal interface material, with the objective of minimizing the interfacial thermal resistance. A fan-cooled heat sink was affixed to the lid. This solution enabled a substantial reduction in the maximum temperature of the 3D stacked package, from 207 °C to 97.3 °C. Furthermore, given the disparate thermal conductivity and thermal expansion coefficients of diverse materials in three-dimensional packaging, numerous thermal stress and deformation problems are likely to arise. In severe cases, the phenomenon can lead to fractures and failures at the connections of each layer. Thermal analysis and research on TSVs have been demonstrated to be effective methods for mitigating these issues [110]. In a seminal study, Liu et al. [111] pioneered a novel approach by integrating research on thermal and signal integrity. They proposed a differentiated TSV structure that exhibits a remarkable capacity to mitigate thermal stress without compromising signal integrity. The large-diameter TSV under a pad was substituted by four identical small-diameter TSVs, and the cross section of the large-diameter TSV was the circumcircle of the four small-diameter TSVs. In comparison with conventional TSV, the peak thermal stress and the occupied silicon area of the differentiated TSV were reduced by 33 MPa and 54 μm^2^, respectively. The utilization of a differentiated TSV as the grounding TSV has been demonstrated to enhance the efficacy of shielding interference from aggressive TSVs. This approach has been shown to result in a 15 dB reduction in crosstalk noise over a frequency range of 3 GHz.

Among advanced packaging solutions for CPO and high-performance optoelectronic integrated systems, FOWLP, TSV, and TGV are currently the three mainstream three-dimensional interconnect and heterogeneous integration technology platforms. Each exhibits distinct advantages concerning signal integrity, thermal management, optical coupling efficiency, process complexity, and cost control, exhibiting clearly delineated application boundaries. A systematic analysis of these differences in structural principles, process implementation, electrical performance, optical compatibility, integration density, and reliability provides valuable guidance for selecting packaging architectures for CPO systems.

From a structural and process perspective, FOWLP is a fan-out packaging technology based on a reconstructed wafer, interconnecting the PIC and EIC via TMVs. This technology is notable for its silicon substrate independence and cost-effectiveness, offering low material costs and excellent warpage control. These characteristics render it particularly well-suited for high-yield, large-scale optoelectronic hybrid integration scenarios. TSVs, in contrast, employ an alternative method to achieve high-density electrical interconnects in the vertical direction. This method involves the etching of deep vias in a high-resistance silicon wafer, followed by the filling of these vias with metal. These vias are frequently utilized in silicon interposers or three-dimensional stacked structures, providing optimal interconnect density and CMOS process compatibility. However, they also encounter challenges, including elevated manufacturing costs and thermomechanical stress. TGVs employ glass as the interposer material, with through-holes formed via laser-induced etching or wet etching processes. These holes are then metallized for electrical connection. Glass, with its low dielectric constant, low loss, optical transparency, and a coefficient of thermal expansion similar to that of silicon, offers unique advantages in CPO, making it particularly suitable for CPO systems that require high bandwidth, low transmission loss, and high-precision optical coupling. With regard to electrical performance and high-frequency characteristics, TSVs are vulnerable to parasitic capacitance, signal crosstalk, and transmission loss in high-frequency environments due to the high dielectric constant and limited resistivity of the silicon substrate. Despite the potential for enhancement of performance through the implementation of high-resistance silicon and supplementary insulating layers or shielding structures, the high-frequency performance of these materials remains constrained. FOWLP utilizes RDLs with a lower dielectric constant, a strategy that has been demonstrated to effectively mitigate signal loss and crosstalk, thereby showcasing remarkable high-frequency transmission characteristics. TGVs benefit from the low dielectric constant and extremely low dissipation factor of glass itself, exhibiting optimal signal transmission performance in high-frequency and millimeter-wave applications. In terms of optical coupling and packaging compatibility, FOWLP supports edge-emitting or surface-emitting coupling by retaining the optical interface at the edge of the PIC and designing an opening structure. The probability of packaging contamination is minimal, and the optical performance exhibits consistency both before and after packaging. The TSV interposer generally necessitates an auxiliary optical coupling structure. However, silicon materials exhibit absorption losses in particular bands, thereby constraining their range of applicability. The high-temperature steps and chemical–mechanical polishing processes inherent to its fabrication may result in contamination or stress damage to the PIC. Due to the full-band light transmittance of glass, TGV is capable of direct integration with waveguides, grating couplers, and edge-coupling structures. The thermal expansion coefficient of the silicon-based optical chips is matched, thereby helping to suppress optical path deviation caused by thermal mismatch and improve coupling accuracy and long-term stability. Furthermore, TGV supports local heating processes, thereby facilitating the repair and replacement of optical modules. In terms of integration density and scalability, TSV offers the highest vertical interconnect density with micron-level through-hole pitch, making it suitable for high-density 3D stacking and silicon interposer integration. Although FOWLP does not fully meet the requirements of TSV in terms of vertical interconnection, it facilitates multi-chip fan-out integration through multi-layer RDL and μbump technology, ensuring excellent parallel scalability and compatibility with optoelectronic heterogeneous systems characterized by medium-to-high integration density. TGV facilitates double-sided wiring and large-area panel-level processing, showcasing exceptional scalability and multi-chip co-integration capabilities. Its use as an interposer for optoelectronic co-packaging, integrating PICs, EICs, lasers, and fiber arrays, is particularly advantageous, as it enables the achievement of high-bandwidth density and low-power system construction. In regard to the management of thermal processes and the assurance of reliability, TSVs have been demonstrated to facilitate optimal heat dissipation, a consequence of silicon’s high thermal conductivity. However, the mismatch in thermal expansion coefficients between the metal filler and silicon can result in thermal stress and warping. FOWLPs exhibit reduced thermal conductivity and generally necessitate integration with thermal interface materials or embedded metal heat dissipation structures. The utilization of plastic encapsulation is instrumental in the mitigation of warping, thereby ensuring optimal thermomechanical reliability. However, the thermal conductivity of glass is limited, which restricts the effectiveness of TGV in dissipating heat. However, their matching thermal expansion coefficient with silicon has been shown to significantly reduce thermal stress, improve the mechanical stability and fatigue resistance of the package, and excel in thermal cycling tests.

In summary, FOWLP, TSV, and TGV are each suitable for different scenarios in CPO system packaging. FOWLP is suitable for medium-to-high-density, low-cost, and high-yield optoelectronic hybrid integration, typically for short-reach interconnects between PICs and EICs. TSV demonstrates particular proficiency in ultra-high-density 3D stacking and silicon-based heterogeneous integration, rendering it well-suited for scenarios that demand extreme interconnect density, such as AI accelerators and HPC. TGV, with its superior high-frequency performance, optical transparency, and thermomechanical reliability, is an ideal optoelectronic fusion platform in CPO systems, particularly suitable for the packaging of 400 G/800 G and higher-speed optical engines. As the TGV process matures and its cost continues to decline, it is expected to become the mainstream CPO packaging path, propelling silicon photonics technology from integration to a new stage of mass production, maintenance, and scalability.

## 3. Optical Interconnect Technology

Active and passive photonic devices built on dielectric waveguide technology are essential core components of CPO architectures, and efficient coupling of optical fibers and waveguides is equally crucial in CPO architectures. The significance of these materials lies in their central role in PIC, a field that has seen rapid advancements in recent years. This central role stems from the inherently compact geometry of the waveguide structure, which enables efficient light field confinement at the micron and even submicron scales. This, in turn, results in significantly enhanced optical intensity relative to bulk materials within a limited physical volume. Concurrently, it is imperative to achieve low-loss, high-alignment-tolerance coupling between optical fibers and on-chip waveguides to facilitate the efficient channeling of external optical signals into and out of the PIC. Achieving high-density integration, strong light field confinement, and efficient optical coupling is imperative for CPOs to accomplish the objectives of ultra-high bandwidth density, low-power interconnection, and tight coupling of optoelectronic chips.

### 3.1. Femtosecond Laser Direct Writing Waveguide

The advent of advanced computing capabilities and the escalating demands for data transmission in the domain of emerging AI have precipitated a surge in research on and development of CPO technology. The pursuit of immersive experiences in augmented reality (AR) and virtual reality (VR) technologies has also contributed to the proliferation of CPO technology, which boasts ultra-high bandwidth density and low-power interconnection advantages [68,112,113]. In this context, the development of transparent screens or substrates that integrate multiple electronic and photonic functional components has become one of the key paths to achieving the next generation of lightweight, highly integrated mobile transparent display devices. This development has attracted widespread attention from the industry and academia. Among the numerous technical solutions for exploring transparent integrated optical devices, FLDW technology has demonstrated unique application potential. The primary advantage of this technology is its capacity to execute 3D micro-nano processing within transparent substrates, encompassing various optical glasses, crystals, and even polymers. It can also “carve” complex photonic structures, such as optical waveguides, grating couplers, and microcavities, with exceptional spatial resolution and adaptability. This non-contact processing feature, with a minimal thermal effect, enables FLDW to directly construct functional photonic circuits and devices inside transparent substrates without significantly affecting the macroscopic optical transparency of the substrate. It provides robust technical support for the development of highly integrated, high-performance transparent photonic subsystems, a crucial element in promoting the application of CPO architecture in transparent display platforms and the miniaturization of optical engines in AR/VR devices.

In the early days, femtosecond lasers were utilized to inscribe waveguides in glass, thereby fabricating optical devices for the telecommunications industry [114]. By focusing the laser beam through a microscope objective, transparent but visible circular elliptical damage lines were successfully written in bulk glass made of high-silicon oxide, borate, sodium calcium silicate, and fluorozirconate. An investigation was conducted into the effects of 810nm femtosecond laser radiation on diverse glass samples. For both pure quartz glass and germanium-doped quartz glass, the refractive index generated by a single laser pass increased by approximately 0.015 and 0.01, respectively, at the center of the damaged area. Following ten laser passes, the refractive index at the core of the damage exhibited a value that was 0.035 higher than that of the surrounding glass in germanium-doped quartz. Optical waveguide bundles, comprising multiple individual waveguide scans, are particularly well-suited for the integration of BG structures [115]. Low-repetition-rate femtosecond lasers are utilized to generate waveguide bundles in bulk glass materials. These waveguide bundles consist of multiple parallel refractive index change scans. Furthermore, the central scan is divided, thereby introducing 840 nm and 1550 nm Bragg grating (BG) structures. It has been demonstrated that a transmission signal spectral loss of >36 dB is achieved at a wavelength of 1550 nm using a fused silica second-order Bragg grating waveguide (BGW). This result corresponds to an intrinsic grating efficiency greater than 16 dB/cm. The waveguide bundle’s diameter can be readily adapted to a broad wavelength range, spanning from 1 to 50 μm. The insertion of the BG structure exerts minimal influence on the transmission characteristics of the waveguide bundle, thereby enabling the BGW to adjust a broad wavelength range in single-mode or multimode optical pathways.

The visibility of photonic elements inscribed in glass is primarily attributable to the scattering phenomenon facilitated by scattering centers. For waveguides, the phenomenon of scattering results in a degradation of loss performance. The presence of any discontinuity, induced by laser irradiation within the glass matrix, has the potential to result in the scattering of the guided light, thereby leading to an increase in propagation loss (PL). However, in the cross-sections of previously reported FLDW waveguides, the dense “light-guiding core” region is usually accompanied by irregular rarefaction regions. These regions are composed of low-density structures and are usually located in the waveguide cladding. It has been established that light leaks into these rarefaction regions through the evanescent field, which results in significant scattering losses [116,117]. The employment of femtosecond lasers in the inscription of invisible photonic elements in glass, namely high-transmittance (HST) waveguides, facilitates a comprehensive, coordinated regulation of the thermodynamic and kinetic behavior of the material fluid within the laser-irradiated confines. This approach enables the precise modulation of the waveguide’s cross-section, thereby suppressing the formation of scattering centers within the waveguide [118]. In comparison with conventional waveguides, the light leakage (illustrated in red, green, and blue coupling light) is remarkably reduced by an order of magnitude, thereby ensuring high transmittance under conditions of bright illumination. The universal dynamic model, predicated on the frozen shock wave diffusion process, finds application in all glass types, irrespective of their composition. The ultra-wide tuning of the HST waveguide mode diameter, ranging from 4.9 μm to 26.5 μm, is achieved, thereby enabling the realization of diverse transparent screens through mode matching with fiber sources and integrated planar waveguides of varying operating wavelengths. The relevant progress is illustrated in Table 3.

**Figure 5:**
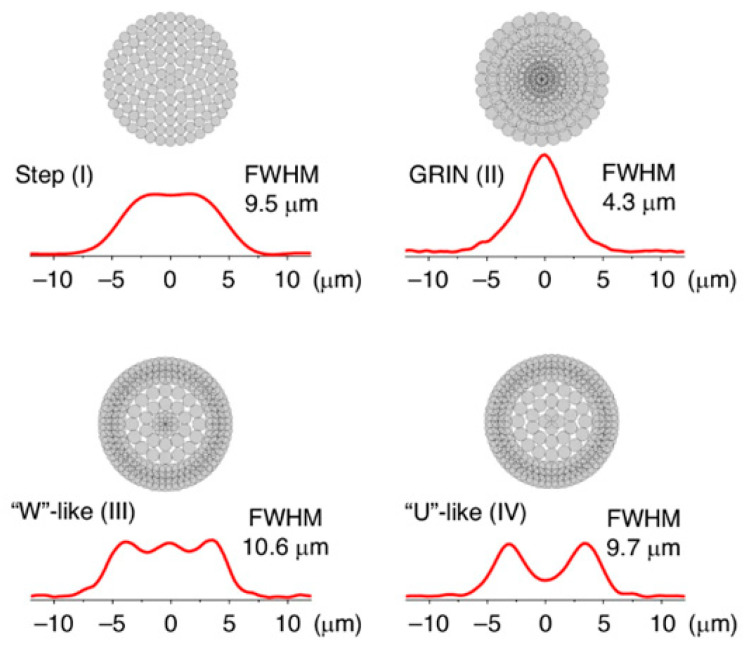
Scan line overlap scheme for four fundamental mode waveguides [119].

FLDW is a reliable technology that can customize the cross-sectional shape of waveguides and allow for flexible transformation along the waveguide cross-section [122,123]. However, irrespective of whether based on traditional planar lithography or contemporary FLDW technology, precise control of the refractive index distribution of waveguides remains elusive, primarily due to the absence of a universal technical means to modify the refractive index with high spatial resolution and spatial selectivity. This has resulted in a substantial impediment to the precise control of mode field distribution and optical coupling, particularly in 3D waveguides. The overlapped controlled multi-scanning (OCMS) method, which is based on laser direct lithography, has been demonstrated to be an effective solution to this problem [119], as shown in Figure 5. The utilization of OCMS technology has led to the development of a manufacturing process for 3D photonic devices, characterized by a complex refractive index distribution. The process demonstrates a capacity for achieving a refractive index modification accuracy of approximately 10^−5^, with the minimum width of the refractive index modification unit being capable of control to 50 nm. This is nearly an order of magnitude lower than the previously reported results. According to the aforementioned approach, variable mode field distribution, robust broadband coupling, and dispersion-free LP21 mode conversion of supercontinuum pulses with a maximum coupling ratio deviation of <0.1 dB over a 210 nm broadband (931 nm−1141 nm) have been achieved. This approach provides a path to achieve ultra-broadband and low-dispersion coupling in 3D photonic circuits, offering significant advantages over conventional planar waveguide-optical platforms and enabling on-chip transmission and manipulation of ultrashort laser pulses and broadband supercontinuums. The relevant progress is illustrated in Table 3.

**Figure 6:**
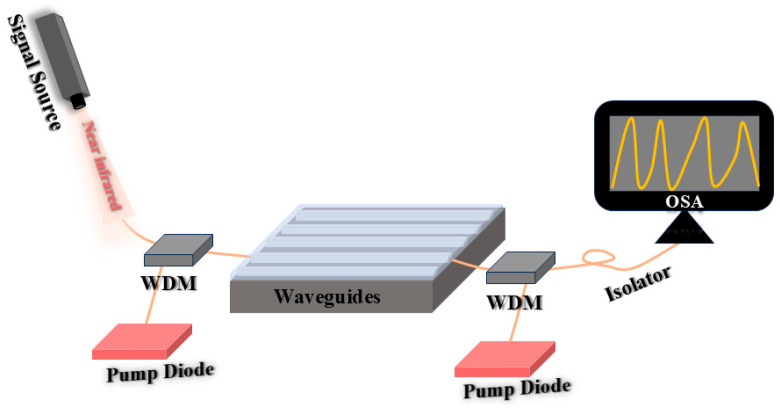
Gain test of a dual-stage pumping configuration.

Optical waveguides prepared in single crystals provide significant active/passive optical elements for photonic integrated circuits. Single crystals exhibit several advantages over amorphous crystals, including reduced optical loss in the visible to mid-infrared spectrum, an enlarged peak emission cross-section, and an increased doping concentration. In the context of rare-earth ion-doped devices, gain represents an indispensable metric, and a dual-stage pumping configuration is frequently employed for experimental testing purposes, as illustrated in Figure 6. However, the utilization of femtosecond laser technology for the fabrication of Type-I positive refractive index modified waveguides in single crystals is confronted with significant challenges. The combination of slit shaping and an oil immersion objective lens with femtosecond laser direct writing technology resulted in the preparation of low-loss Type-I waveguides in single crystals [120]. This method enables precise control over the geometry, dimensions, mode field, and refractive-index distribution of the waveguide, with a spatial resolution of up to 700 nanometers and a positive refractive-index variation of up to 10^−2^. This introduces new degrees of freedom for the design and preparation of active/passive optical waveguide devices. As a proof-of-concept, the present study successfully fabricated a 7 mm long circular gain waveguide in an Er^3+^-doped YAG single crystal. The waveguide demonstrated a low transmission loss of 0.23 dB cm^−1^, a gain of approximately 3 dB, and polarization insensitivity. The newly developed technique is theoretically applicable to any single crystal and has broad application prospects in integrated optics, optical communications, and photonics.

Gallium-rich barium gallium germanium (BGG) oxide glass is a suitable material for waveguide fabrication using FLDW because it always has a positive refractive index change. However, as an oxide glass, untreated precursors will result in significant retention of hydroxyl groups in the glass melt. This is deleterious to Er^3+^ emission and can also lead to significant luminescence quenching of other rare earth elements, such as thulium [124]. In the context of thulium-doped (Tm^3+^) BGG glass, a notable advancement was achieved through the fabrication of an optical waveguide exhibiting a nearly circular cross-section. This accomplishment was realized through the implementation of a singular scan employing femtosecond laser direct writing technology. A significant milestone was marked by the attainment of laser output based on this waveguide, a feat that has not been previously accomplished [121]. The waveguide demonstrated a positive refractive index change and minimal propagation loss. Structural analysis demonstrated that the laser-induced refractive index increase was predominantly attributable to the formation of highly polarized non-bridging oxygen, as opposed to ion migration or densification. The researchers constructed a laser resonator composed of dichroic mirrors, and pumped it with a 1.6 µm erbium-doped fiber laser. In the experiment, an output coupling mirror with a 17% transmittance was utilized, resulting in a single-mode laser output of up to 62 mW at a wavelength of 1.89 µm. The experimental outcomes demonstrated a slope efficiency of 17%, a beam quality factor of M^2^ ≈ 1.2, and a lasing threshold (absorbed pump power) as low as 54 mW. A comparison of this single-scan waveguide laser to bulk thulium-doped BGG lasers and conventional materials, which require multiple scans or complex cladding structures, reveals significant advantages in simplified fabrication, reduced processing time, and high beam quality. This work demonstrates the potential of BGG glass as an excellent platform for the rapid fabrication of mid-infrared integrated photonic devices. Its waveguide properties are compatible with standard optical fibers, facilitating system integration and paving the way for subsequent exploration of applications such as erbium-doped 2.8 µm lasers.

### 3.2. Ion-Exchange Glass Waveguide

The incorporation of silicon-based photonic transceivers necessitates the development of innovative packaging methodologies and materials to achieve substantial enhancements in power efficiency and manufacturing [125]. Glass core substrates with integrated optical interconnects made of silver ion exchange provide low-loss and high-density fiber-to-chip coupling. Furthermore, they facilitate the transition to high-throughput manufacturing through the implementation of panel-scale processing and photonic flip-chip assembly. A significant application of CPO is the packaging of switch integrated circuits with data throughputs ranging from tens to hundreds of terabits.

Glass waveguides prepared by ion exchange have been considered one of the most promising integrated optical devices due to their compatibility with optical fibers, low cost, low loss, ease of high-concentration rare earth ion doping, ease of integration, and good environmental stability. Consequently, since the initial discovery of Ti^+^ ion exchange waveguides in silicate glasses containing sodium and potassium oxides in the 1970s [126], extensive research has been conducted on the preparation of glass waveguides using ion exchange technology. The fabrication of glass waveguides through ion exchange typically necessitates the utilization of electric field assistance, thereby rendering the inevitable occurrence of Joule heating a perpetual challenge. During the field-assisted ion diffusion process, the temperature of the glass wafer increases in synchrony with the flow of current [127]. When a silicon dioxide wafer of equivalent thickness is substituted for the glass wafer and the same voltage is applied to the silicon dioxide wafer, the current amplitude observed is three to four orders of magnitude lower than that of the glass wafer. The extent of the temperature increase of the glass wafer is determined by the competition between heat generation and heat dissipation. As the temperature of the glass wafer rises, its capacity for both heat generation and dissipation is augmented. Research has demonstrated that the Joule heating effect exerts a substantial influence on the waveguide manufacturing process, encompassing the stability of ion diffusion, the theoretical modeling of the ion diffusion process, and the depth uniformity of the waveguide on the glass substrate. The polarization effect of the polarizer is based on the attenuation difference between two orthogonal polarization-guided modes [128]. The embedding depth and width of the ion-exchanged glass waveguide in the graphene/glass hybrid waveguide are optimized to 2.6 and 11.5 μm, respectively. In the domain of telecommunications, the polarization extinction ratio of the polarizer is 27 dB when the length of the graphene coating along the propagation direction is 4 mm. When the PMMA film undergoes spin-coating on the waveguide, the ER of the PMMA-coated waveguide increases by less than 1 dB. However, for the PGGW, the transmission loss of the two orthogonal polarization modes differs by 27.3 dB. Furthermore, the polarization extinction ratio can be enhanced by decreasing the chemical potential of the graphene film. This configuration may enhance the light transmission efficiency in CPO.

The process of embedding an optical waveguide in a photothermorefractive glass (PTR) involves two distinct mechanisms: thermal ion exchange (TIE) and subsequent field-assisted ion migration (FAIM) [129]. A buried optical waveguide with a core diameter of 8.7 μm × 9.0 μm and a leading edge located approximately 12.5 μm below the surface of the glass substrate was obtained. The insertion loss of the prepared channel waveguide was measured by butt-coupling two single-mode optical fibers to the two ends of the optical waveguide chip to be tested. The transmission loss of the optical waveguide and the coupling loss between the waveguide and the single-mode fiber were determined by the backoff method. Utilizing an operating wavelength of 1550 nm, the data was fitted, resulting in a transmission loss of 0.08 dB/cm for the waveguide. The coupling loss with the standard single-mode fiber was measured to be 0.47 dB/facet, thereby demonstrating the feasibility of low-loss integrated photonic devices.

Glass core substrates for integrated optical interconnects made of silver ion exchange provide low-loss and high-density fiber-to-chip coupling. In order to achieve high-performance and reliable glass-based photonic packaging in data centers, AI computing clusters, high-performance computers, or 6G applications, optimized glass that meets both requirements is necessary. Corning proposed ultra-low-loss ion exchange waveguides in optimized alkali glass for co-packaging [130]. Corning has proposed a large optical path for the manufacturing of multi-chip components, with an area of 75 mm × 75 mm. Additionally, they have proposed the use of IOX waveguides for optical routing, with an area of 255 mm × 420 mm. The selection of the hot ion exchange process for waveguide preparation was made on the basis of the benefits of batch processing of multiple glass substrates and the scalability of panel-level processing. The peak refractive index exhibited an increase of 4.7 × 10^−3^, and the waveguide loss was measured to be 0.034 dB/cm at a wavelength of 1310 nm. The coupling loss at the fiber–waveguide interface was 0.31 dB, which is consistent with the simulation results. At an operating temperature of 85 °C, the waveguide demonstrated stability over five years, exhibiting no degradation, thereby preserving the integrity of the optical signal. The efficacy of alkali glass and SiO_2_ barrier layers in meeting the high-temperature requirements of CPO applications, while overcoming the reliability risks of alkali migration into attached components, has been demonstrated. In the case of a SiO_2_ layer thickness of less than 200 nm, evanescent coupling was successfully achieved between the Si_3_N_4_ waveguide of the photonic integrated circuit (PIC) and the SiO_2_ layer. This coupling resulted in a loss of less than 0.5 dB.

The augmentation of capacity in wireless access networks is an impending trend; nevertheless, the heightened power consumption that pluggable transceivers must grapple with is a formidable challenge. At the OFC conference, Corning and Ericsson put forward a pioneering proposal for an integrated glass waveguide circuit for CPO in wireless access networks [131]. In order to address the complex problem of optical fiber assembly, Corning designed a 2 × 4 MIMO waveguide optical circuit. This design achieved star/mesh interconnection of seven optical ports (48 waveguides), thereby replacing traditional optical fiber bundles. Single-mode waveguides are fabricated in large-size glass using Ag ion diffusion, and the mode field mismatch loss is a mere 0.3 dB/interface. When tested in the 1310 nm band and with the 50 mm length, it was found that the straight waveguide only produced a 1 dB loss. Concurrently, the mean loss between the two ports and the loss between the connectors were 2 dB and 0.8 dB (including the mode field mismatch loss of 0.3 dB). The optical circuit is engineered to withstand extreme temperatures ranging from −40 °C to 125 °C, making it well-suited for the demanding environments found in wireless base stations. A multi-layer bonding process is employed to adhere a 0.7 mm-thick glass substrate to a 16-layer Panasonic Megtron 7 PCB, thereby ensuring the accommodation of the RF board’s precise layout. The bandwidth density has been demonstrated to reach 2 Tb/s/cm^3^, marking a more than 100-fold increase and demonstrating a capacity to meet the 6 G MIMO requirements. The integration of Corning’s glass photonics platform and Ericsson’s communication system architecture has enabled the large-scale implementation of optical interconnect technology in wireless base stations.

The imperative to reduce the length of package interconnects and mitigate electrical losses has persistently been a pressing problem to be addressed. As the bandwidth requirements of next-generation solutions increase, the number of optical fibers that must be managed within the rack chassis will increase concomitantly. The introduction of panel-level optical circuit boards has the potential to simplify this process. Corning’s initial production of a board-level fan-out optical path measuring 420 mm × 255 mm and with a thickness of 0.7 mm served as a replacement for optical fibers between the CPO transceiver and the optical connector at the panel [132]. The glass waveguide circuit layout consists of 16 waveguide groups, with a total of 1024 optical interconnects. Each waveguide group contains 64 ion-exchange waveguides, with a minimum pitch of 50 μm. The process flow is employed to streamline the waveguide manufacturing process for large panels. The dimensions of the area in question, from the panel to the CPO area, are 75 mm × 75 mm. This area is located centrally within the glass panel, which has dimensions of 420 mm × 255 mm. The 16 waveguides within each group are separated by 250 μm to ensure compatibility with standard MPO-16 connectors at the faceplate. This optical circuit provides 1024 waveguides extending from one edge to the CPO region, exhibiting a measured transmission loss of 0.1 dB/cm and less than 0.04 dB/cm at a wavelength of 1310 nm. The relevant progress is illustrated in Table 4.

The manufacturing process itself is a key factor in ensuring optimal performance and integration density, given the significant advancement of data transmission technology. However, the prevailing manual methodologies for the characterization of these waveguides impose limitations on scalability and engender inefficiencies in the fabrication of high-speed data transmission systems. An automated high-speed characterization system for ion exchange waveguides on large glass substrates can effectively solve the aforementioned problem [133]. The light intensity peak positioning is automatically optimized through a two-stage spiral scanning process involving a cooperative movement of the optical fiber and photodiode. This process, known as coarse-fine spiral scanning, reduces the time required for single waveguide alignment to 60 s. Concurrently, the integrated machine vision system, operating on the basis of the DXF layout file, automatically identifies 1053 waveguides and positioning marks on a 303 × 227 mm glass substrate, with a positioning accuracy of ±0.13 dB. Furthermore, the full-process automation architecture is adequate to support the continuous measurement of 27 cm long waveguides (complete board testing in 18 h), which is three times more efficient than manual inspection. The system has been demonstrated to accurately identify an abnormal waveguide, and its fault location is consistent with manual retest results. This provides key technical support for mass-production quality control of high-density glass waveguide panels in CPO. This technology is compatible with femtosecond laser direct writing waveguides, which has been demonstrated to accelerate the process development cycle for waveguides with complex parameters. This is expected to address the bottleneck of waveguide performance testing in future CPO mass production.

### 3.3. Fiber Coupling

The realization of CPO necessitates innovation and breakthroughs in multiple dimensions, ranging from materials and devices to photonic chips and packaging integration. Multi-channel fiber IO is a foundational technology in this field [134,135,136,137]. In the context of CPO, the optical signal in the optical fiber must be transmitted to the waveguide of the PIC, a process that may involve waveguide–fiber coupling and waveguide–waveguide coupling. Coupling loss is a significant metric in the field of optical coupling, which is comprised of three primary components: alignment deviation loss, Fresnel reflection loss, and mode field mismatch loss. Alignment deviation loss, on the other hand, is caused by the misalignment of the end faces of the waveguide and the optical fiber. The relative position of the optical fiber and the waveguide can be improved through the use of positioning grooves or the design of high alignment tolerance coupling structures. Fresnel reflection loss is attributable to the optical reflection of the end face, resulting from the disparity in refractive index between the waveguide and the optical fiber materials. The efficacy of the coating can be enhanced through the application of an anti-reflection film, in conjunction with the incorporation of a refractive index-matching liquid. Mode field mismatch loss, or MFM loss, is caused by the mismatch between the size of the optical fiber and the size of the waveguide. The optical fiber size is approximately 10 μm, and the waveguide size is nearly submicron, resulting in a mode spot mismatch problem. The mode spot can be matched by designing a coupler.

In the context of high-capacity, low-power data centers, the implementation of mode division multiplexing (MDM) technology is anticipated to result in a notable augmentation in the number of parallel channels transmitted within a single waveguide and optical fiber. This development is expected to enhance the capacity of optical fiber interconnection [138]. Multimode chip–fiber couplers, an indispensable component, have been the focus of extensive research; however, challenges persist in multimode interfaces [139,140]. Edge couplers have been demonstrated to be an effective solution to this problem [141]. The dual-mode fiber-chip edge coupler, which is based on silicon photonic integration, has been designed to support 2 × 100 Gbps/λ MDM optical interconnection. The device’s compatibility with standard CMOS processes and its compact size are notable advantages. Additionally, it facilitates efficient coupling of the fundamental mode in the chip waveguide to the few-mode fiber (FMF), thereby addressing the issue of large mode field mismatch between silicon-based chips and optical fibers. In a 2 × 100 Gbps PAM4 MDM transmission configuration over 40 m of dual-mode fiber, the bit error rate of each channel is observed to meet the 7% forward error correction threshold. This provides a key integrated multimode interface solution for high-density, high-capacity optical interconnects, particularly well-suited for data centers requiring ultra-high bandwidth and low power consumption. Future performance enhancements will necessitate the optimization of taper tip processing and fiber mode field matching.

Mode division multiplexing technology has undergone gradual development in the domain of fiber–chip–fiber optical interconnection, primarily attributable to the inefficient mode coupling between few-mode fiber and multimode photonic chip. In the field of optical communication, several integrated schemes have been proposed. These schemes are based on vertical coupling and edge coupling, to achieve multimode fiber–chip coupling. The integrated multimode coupler, employing a singular vertical grating coupler or edge coupler, is ordinarily constrained by the number of guided modes processed. Conversely, the implementation of a 2D vertical grating coupler array will result in substantial insertion loss. The multimode coupler, composed of a tapered few-mode fiber and a silicon integrated multi-level waveguide cone, facilitates direct coupling between high-order fiber modes and high-order waveguide modes, exhibiting high coupling efficiency and low crosstalk based on the edge coupling scheme [142]. The six linear polarization modes in the few-mode fiber are coupled with the integrated waveguide with low insertion loss. Then, the mode evolution principle is used to convert the input mode into the desired waveguide mode with low mode crosstalk. The coupling efficiency exceeds 87%, while for the mode conversion process, the efficiency surpasses 99%, and the mode crosstalk is reduced to below −25 dB. The excellent performance achieved may open new horizons for different mode division multiplexing applications, thereby realizing effective capacity expansion in fiber–chip–fiber optical interconnects and optical communication systems. The silicon-integrated multi-level waveguide taper embedded in the polymer waveguide can achieve efficient mode conversion from the input fiber mode to the desired waveguide mode based on the principle of mode evolution, which provides a certain foundation for future polymer waveguide-assisted CPO performance improvements.

The incorporation of a multi-stage tapered structure, augmented by nanogroove waveguides and SU8 polymer upper cladding waveguides, has been demonstrated to effectively mitigate the mode mismatch issue that arises between FMF and the multimode silicon waveguide [143]. The coupler facilitates the direct conversion of six linear polarization modes to on-chip waveguide modes. The device utilizes a tapered FMF to compress the mode field, then matches the polymer waveguide mode, and finally achieves low-loss mode evolution in a three-stage conversion structure to the silicon nanotip. This approach effectively avoids the bandwidth limitation of traditional grating couplers. In the C-band, it attains a mode conversion efficiency of >98%, a crosstalk of <−19 dB, and a total coupling efficiency of >85%. The 6 × 6 μm^2^ polymer waveguide solution exhibits an LP010 mode coupling efficiency that exceeds 94%. The SOI process, which is based on electron beam lithography (EBL) and ICP etching, renders the SU8 waveguide superfluous for the etching process and is compatible with CMOS processes. The provision of essential technical support for fiber–chip interconnection in MDM–WDM hybrid multiplexing is anticipated to enhance the transmission capacity of co-packaged optical systems.

Given the rapid growth in data-center traffic, which is doubling every 2–3 years, Ethernet switches must be capable of supporting a throughput of 51.2 Tb/s and above. Additionally, pluggable optical modules are facing limitations in power consumption and front panel bandwidth. CPO has emerged as a groundbreaking solution through optoelectronic co-packaging. However, its implementation must address the challenges of achieving multi-channel fiber I/O compatibility with reflow soldering [144,145]. Traditional grating couplers have been shown to exhibit limited bandwidth and polarization sensitivity. Conversely, edge couplers have been demonstrated to necessitate substrate thinning, a process that can compromise the structural integrity of the device. Both of these methods rely on permanent fiber bonding and are incompatible with the elevated temperatures associated with reflow soldering. Existing detachable solutions have been shown to exhibit high levels of loss and difficulty in meeting the requirements [146,147].

The expansion of the beam edge coupling facilitates the implementation of a detachable low-loss fiber coupling interface for CPO, thereby addressing the fiber I/O challenge posed by CPO modules in the context of the reflow soldering process [148]. The development of a detachable coupling interface for the extended beam edge coupling can seamlessly support many channels with high efficiency. The permanent chip end connector (EBPC), which incorporates a microlens array and an MT positioning connector, and the detachable fiber end connector (EBFAC) form a dual-component architecture. This architecture utilizes beam expansion to convert the waveguide mode into a 160 μm beam spot, thereby significantly relaxing the alignment tolerance. Concurrently, the infrared imaging sensor is capable of calibrating the beam angle of the EBFAC, as well as aligning and solidifying the EBPC and the connector in an active assembly manner. The coupling loss between the SiP chip and the fiber is less than 1 dB, and the loss fluctuation is less than 0.3 dB when the lens is offset by 35 μm. The laser–fiber coupling efficiency is more than 90%, and the loss fluctuation is less than 5% after 20 repeated plugging and unplugging. The replacement of the positioning connector with a ceramic material enables adaptation to the reflow soldering process and facilitates high-density integration of the CPO module. The detachable coupling interface developed by the detachable coupling interface is a promising advancement due to its low coupling loss, ability to support high-density multiple parallel ports, and potential for full solder compatibility. This interface provides a solution for achieving low-loss, high-density, reflow-compatible CPO optical interconnects.

The advent of generative AI has precipitated a marked escalation in data-center traffic. CPO has emerged as a pivotal solution for mitigating power consumption and augmenting bandwidth by reducing electrical interconnect distances. However, the integration of CPO into optoelectronic co-packaging presents challenges, and the development of optical connectivity must address the limitations of direct coupling between PIC and optical fiber [32]. As indicated in the relevant literature, traditional edge coupling is subject to substantial losses resulting from bonding errors [149]. Conversely, evanescent coupling necessitates millimeter-scale tapering to augment chip size.

Typical flip-chip bonding techniques result in positional misalignment of approximately ±3 μm, which can lead to a significant optical coupling loss. A vertically coupled beam expansion lens (VCBEL) was introduced to amplify the alignment tolerance, resulting in a significant reduction in optical coupling loss [150]. Utilizing a 3D-printed prototype, we have demonstrated optical coupling between the PIC and the glass waveguide, exhibiting a typical loss of 1.6 dB and an excess loss of less than 0.1 dB for a misalignment of ±3 μm. Additionally, while the roughness on the glass grooves leads to substantial optical losses in air, our findings demonstrate that the incorporation of a resin lens can markedly mitigate these losses. Moreover, given its properties that are conducive to large-scale integration and the coexistence of wires and optical waveguides, it is anticipated that the glass substrate can also be utilized in large-scale photonic electronic integration platforms. The VCBEL expanded beam coupling scheme is designed to achieve high-tolerance, low-loss flip-chip optoelectronic synchronous integration, providing a high-density, detachable optical I/O solution.

The 3D mode size converter, in the absence of additional lithography, has been demonstrated to facilitate mode conversion on the lithium niobate (LNOI) platform [151]. The height gradient from 600 nanometers to 175 nanometers in the local area of the wafer is achieved by dry etching with a silicon external mask, and the width-tapered structure defined by lithography is combined to form a three-dimensional adiabatic mode conversion waveguide. The mask gap is employed to attain a smooth slope, thereby facilitating local area etching. Subsequent to this, global electron beam lithography and ICP-RIE etching are carried out. Ultimately, a 2.5 μm PECVD SiO_2_ cap layer is deposited. The optical performance of the material is optimized by annealing, a process that involves heating and cooling a material in a controlled environment to achieve a specific crystal structure. The experiment was conducted under a lens fiber with a spot diameter of 2.5 μm. The TE/TM mode coupling loss was measured at a wavelength of 1550 nm to be 1.16 dB/facet and 0.71 dB/facet, respectively. This design effectively addresses the efficiency loss that arises from the leakage of optical modes to the substrate in conventional single-layer inverted tapered structures. Notably, it eliminates the necessity for chemical mechanical polishing or multi-layer lithography, enhancing the simplicity and reliability of the process. The process is compatible with wafer-level manufacturing. This method can be extended to other material platforms, laying the foundation for efficient fiber array packaging for nonlinear and quantum photonics and providing efficient and robust edge coupling solutions for LNOI and other platforms.

In the context of on-chip interconnects, device interfaces demonstrate variations in properties such as refractive index distribution, spatial positioning, dimensions, and orientation. To address the mismatches in size, mode field, and spatial layout among multi-material optoelectronic devices, photonic wire bonding (PWB) technology has emerged [152]. PWB technology utilizes a femtosecond laser direct writing process of photoresist to fabricate polymer waveguides. This method utilizes two-photon polymerization, a process that enables direct optical interconnection between different optical interfaces. Polymer waveguides fabricated using PWB technology exhibit excellent scalability and thermal stability. Through material selection and process optimization, combined with advanced interface inspection and image recognition technologies, repeatable processing of photonic wires between 100 silicon waveguides can be achieved, with an average processing time of 30 s per wire. The measured ensemble of 100 PWB bridges exhibits an average insertion loss of 0.73 dB and a standard deviation of 0.15 dB. Following 225 temperature cycles ranging from −40 °C to 85 °C, the material structure and composition remained intact, exhibiting no performance degradation [153]. In contrast to diffraction or refraction, optical reflection is inherently wavelength agnostic and incurs vanishingly low optical loss in the total internal reflection regime. The reflectors function to redirect and shape the waveguide output, thereby enabling high-density surface-normal coupling to 2-D fiber arrays with low losses and high tolerance to misalignment. Yu et al. [154] reported a universal photonic coupling scheme based on free-form micro-optical reflectors. The micro-optical reflectors were fabricated using two-photon polymerization technology, enabling subwavelength resolution for precise definition of free-form micro-optical elements. At a wavelength of 1550 nm, the coupling loss was measured to be as low as 0.5 dB, and ultrawide band operation was achieved with a 1 dB bandwidth that extended up to 300 nm. The geometric fidelity and coupling efficiency of the couplers were not compromised by heat treatment up to 250 °C. Conventional grating couplers typically exhibit limited coupling efficiency, primarily constrained by the imperfect directionality of the diffraction process. A comparison of silicon and silicon nitride reveals that the former has a lower refractive index contrast, which presents both advantages and challenges for grating couplers. Silicon nitride grating couplers have demonstrated the capacity to achieve an enhanced bandwidth; however, the low refractive index contrast generates diminished light scattering, thereby constraining the coupling efficiency of single-etched silicon nitride gratings [155]. Lomonte et al. [156] proposed a flexible strategy for realizing efficient grating couplers on low-refractive-index platforms. In order to surmount the limitation of reduced scattering intensity, an apodized grating coupler was conceived to display a negative diffraction angle. In order to achieve high directionality in the diffraction process, a metal back reflector was realized beneath the buried oxide layer by means of cryogenic deep reactive ion etching of the silicon handle. The grating coupler demonstrated a coupling efficiency of up to 88% in the telecom C-band, indicating its effectiveness in this frequency range. The relevant progress is shown in Table 5.

## 4. Artificial Neuromorphic Optical Interconnects

CPO technology integrates optical computing engines with electrical switching chips, thereby achieving a substantial reduction in data-transmission power consumption. However, the field faces significant challenges in the realm of intelligent system-level control. Optical components have been demonstrated to exhibit heightened sensitivity to temperature fluctuations, process variations, and driving conditions. Consequently, their operating characteristics may undergo dynamic drift in response to environmental changes. Conventional centralized control solutions that rely on general-purpose processors are plagued by slow response times, low energy efficiency, and an inability to satisfy the demands of concurrent, real-time control of multiple channels. This has emerged as a significant impediment, impeding the effectiveness and dependability of CPO systems. Neuromorphic computing, which draws on the information processing mechanisms of the biological nervous system, offers a revolutionary solution to this control dilemma. The architecture is event-driven, highly parallel, and integrated, and it is particularly well-suited to handling asynchronous and sparse control events (such as real-time bias point calibration, dynamic wavelength locking, multi-channel power balancing, and fault monitoring) across the vast number of optical links in CPO systems. The device has been demonstrated to attain nanosecond-level responsiveness while operating on an ultra-low microwatt power consumption. Consequently, this reduces the energy consumption and latency overhead associated with the control functions themselves.

The exponential growth of data-center traffic, driven by artificial intelligence and machine learning, has precipitated the development of optical interconnect solutions. Silicon-based photonics has become an indispensable component of optical interconnects due to its high-density integration capabilities and compatibility with CMOS processes, especially for the large-scale integration of next-generation high-density interconnects [157]. Silicon-based PICs almost always coexist with EICs, which primarily implement E/O and O/E conversion of end-to-end data, as well as bias, control, and compensation for temperature and process deviations. Photons are well-suited for information transmission due to their non-interacting nature, whereas electrons, due to their repulsive interaction, are particularly well-suited for use in switches and computing elements. This interaction results in transistors consuming significantly less power during switching, thereby providing gain and high precision, while exhibiting dimensions several orders of magnitude smaller than those of photonic devices. Conversely, photonic devices exhibit reduced frequency-dependent losses during the transmission of data over extended distances, facilitate lower latency through asynchronous and repeater-free data movement, and enhance parallelism of high-speed data through optical waveguides. In instances where data is already present in the optical domain, the exchange or processing of photonic signals can become a viable option [158,159]. In silicon-based photonic devices, MRR embedded in PN junctions as modulators has attracted considerable research attention due to their compact size, enhanced power efficiency, and simplified manufacturing that avoids germanium epitaxial growth [160,161]. These advantages position MRRs as a superior alternative to MZI modulators, particularly for large-scale integration in next-generation high-density interconnects. Despite the demonstrated potential for silicon-based MRR in various applications, significant challenges persist in its commercialization. The issue of minor variations in the manufacturing process resulting in substantial alterations to the resonant wavelength is attributable to the high susceptibility to ambient temperature. Consequently, precise post-manufacturing tuning methodologies are imperative [162]. Conventional methods typically employ integrated thermal phase shifters to calibrate the MRR and adjust its resonant wavelength to the target value. However, this approach is accompanied by substantial static power consumption, thermal crosstalk, and volatility issues, which become particularly pronounced as the integration scale increases [163,164,165]. To address this challenge, non-volatile artificial neuromorphic photonic systems have emerged. Non-volatility is a fundamental characteristic of modern inherited photonic systems, as they are key technologies for next-generation data centers, optical neural networks, and quantum information processing. The essential component of a non-volatile PIC is an MRR, which enables independent control of coupling and phase. The programming of these MRRs necessitates meticulous local tuning and the absence of a static energy “set-and-forget” reconfiguration to preserve the programmed state, a prerequisite for achieving truly non-volatile PICs [166,167,168].

**Figure 7:**
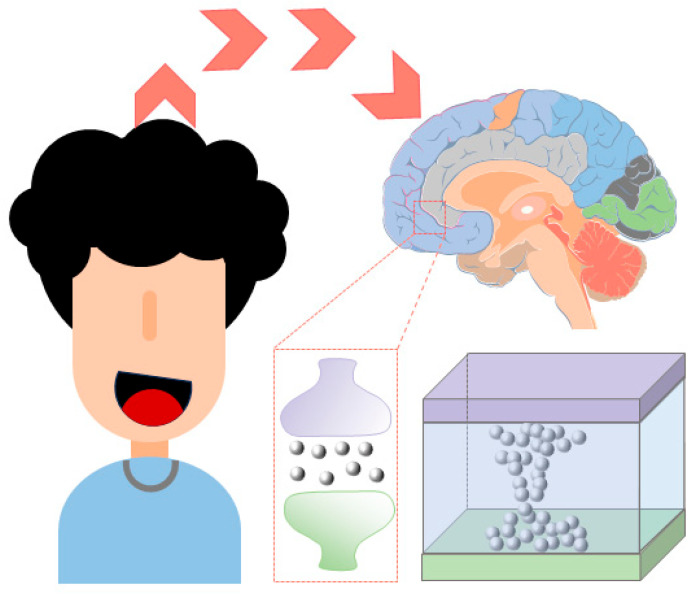
Schematic diagram of a biological neural network and an artificial neuromorphic device.

The central focus of artificial neuromorphic devices as a switching memory device is their capacity to dynamically simulate the fundamental functions and plasticity mechanisms of biological synapses. This capacity provides a physical foundation for constructing efficient neuromorphic computing systems. The fundamental operational principle of these devices is predicated on the controllable modulation of their internal resistance state (resistance change) between a high resistance state (HRS) and a low resistance state (LRS) under external electrical pulse stimulation, accompanied by the generation of set or reset current peaks. This transition in resistance state does not correspond to a simple binary flip; rather, it can be continuously and gradually adjusted, thereby directly simulating the dynamic change process of biological synaptic weights. The conductance value of artificial neuromorphic devices can be regarded as the weight of artificial synapses. An increase in conductance corresponds to an increase in the excitation of synaptic connections, while a decrease corresponds to inhibition, thereby enabling the device to integrate information and store weights. This inherent plasticity renders it a highly promising candidate for simulating biological synapses [169,170], as illustrated in Figure 7.

The essential characteristics of artificial neuromorphic devices that emulate synaptic functions have been distinctly delineated and actualized in research. These features are indicative of the time dependence and activity dependence of biological synaptic information processing, including paired pulse facilitation (PPF), short-term plasticity (STP), long-term plasticity (LTP), and spike rate-dependent plasticity (SRDP) [171,172]. The hardware implementation of these plasticity rules enables artificial neural networks (ANNs) constructed from such synaptic devices to perform complex, brain-like information processing tasks, such as pattern recognition, associative learning, and adaptive control, playing an irreplaceable core role in neuromorphic computing. The intrinsic physical mechanism that drives this resistive state switching and plasticity simulation is usually dominated by the redistribution and migration of ions within the device. The concept of artificial neuromorphic devices is predicated on the premise that ion movement is achieved through electrochemical processes within the nanoscale storage layer. This movement of ions is facilitated by the presence of ion channels in biological neural systems, such as voltage-gated calcium channels (CaVs), which play a pivotal role in the triggering of neurotransmitter release and synaptic excitation. When a voltage pulse is applied, the nanostructure can generate a strong local electric field even at a small voltage drop. The application of an electric field serves to facilitate the oxidation, reduction, and directional migration of cations (e.g., metal ions) or anions (e.g., oxygen vacancies) within the device. This ion migration process has been shown to induce significant alterations in the device’s microstructure, including the formation or dissolution of conductive filaments, and to result in a corresponding change in overall conductivity. These alterations serve to complete the reversible switching between HRS and LRS, as well as the dynamic updating of weights [173,174].

The switching mechanism, which is based on ion dynamics, endows this seemingly simple device with exceptional performance. These inherent properties directly translate into significant application advantages. Firstly, the non-volatility of the resistive state facilitates the storage of learned weight information over an extended period, a process that is critical for the establishment of persistent memory and learning. Secondly, this mechanism naturally supports high-density integration, as individual artificial neuromorphic device units are compact, and their resistive state switching alone represents the stored information and synaptic weights, thereby eliminating the need for additional transistors. Thirdly, the ion migration process generally exhibits a low energy threshold. When combined with nanoscale effects, this configuration enables the device to operate at low voltages and currents, thereby achieving minimal power loss. This is of paramount importance for the development of large-scale, low-power neuromorphic chips, as it directly addresses the energy-efficiency limitations of conventional von Neumann architectures. The collective impact of these exceptional integrated properties has been instrumental in propelling the rapid development and widespread application of artificial neuromorphic devices in neuromorphic computing and revolutionary photonic systems. These developments have yielded innovative hardware-level solutions that have the potential to overcome the current computing power and energy-efficiency bottlenecks in photonics.

The non-volatile implementation of photonic memory constitutes a fundamental challenge in the development of efficient optical computing systems, representing a core component of PIC. Conventional MRR and electro-optical modulators have proven challenging in meeting the requirements of non-volatile storage, a prerequisite for realizing low-cost, long-term stable photonic memory [175,176]. Phase-change material (PCM)-based photonic memory has emerged as a breakthrough solution due to its distinctive bistable properties. PCM can undergo a reversible phase transition between an amorphous state (characterized by high resistance and low loss) and a crystalline state (marked by low resistance and high loss) through thermal or optical excitation, while concurrently maintaining a high switching speed. In conventional PCM systems, germanium antimony telluride (GST) has been the subject of extensive research due to its substantial optical parameter contrast [177,178,179]. However, its intrinsic absorption coefficient in the amorphous state is excessively high, which complicates the fulfillment of the requirements for large-scale photonic networks. This is particularly evident in applications such as deep neural networks, where multi-layer photonic architectures necessitate core memory to achieve ultra-low optical loss. The material properties of GST act as a fundamental limitation in this regard.

**Figure 8:**
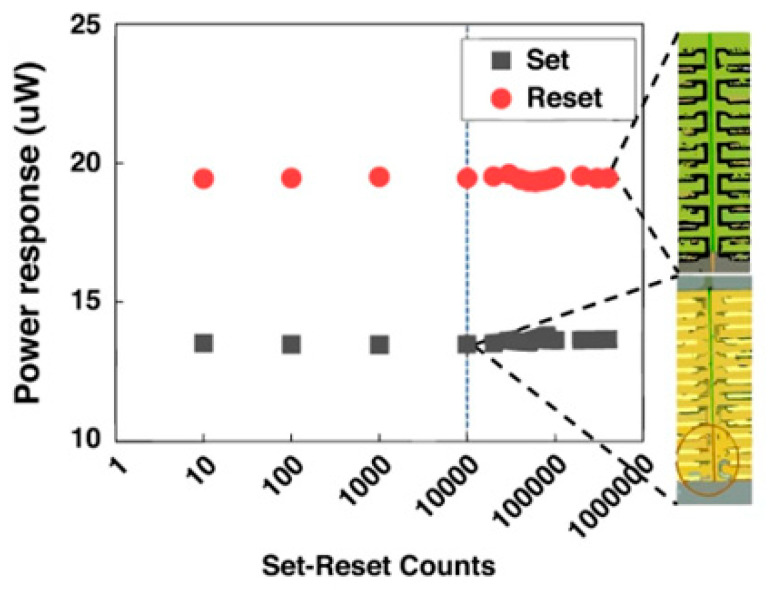
Two-state photoresponse changes over 500,000 switching cycle [180].

A multi-level non-volatile photonic random access memory (P-RAM) with programmable electrical functionality has been developed. This novel device is based on a new phase-change material, Ge_2_Sb_2_Se_5_ (GSSe), and provides a foundational device for the development of artificial neuromorphic light engines [180]. The device utilizes the key feature of GSSe’s extremely low optical absorption coefficient (2.0 × 10^−5^) at a wavelength of 1550 nm in the amorphous state to achieve ultra-low optical insertion loss (the total loss of a 4-bit unit is only 0.12 dB), which is significantly better than traditional GST-based photonic memory. The design of a double-sided tungsten–titanium microheater, with a 500 nm separation from the waveguide to balance thermal efficiency and optical loss, and the use of an aluminum oxide passivation layer for protection, has been optimized. As a result, this P-RAM has been shown to achieve an amplitude modulation efficiency of up to 0. The material demonstrates a 2 dB/μm performance while exhibiting optimal signal modulation capabilities. Its reliability is evidenced by the successful completion of a stable write–erase cycle test of more than 500,000 times, a durability that significantly surpasses that of analogous devices, as illustrated in Figure 8. The device’s multi-state operation exhibits an energy consumption of approximately 1.5 nJ/dB, while its capacity for 4-bit (16-state) uniform quantization is supported. Notably, the device’s maximum extinction ratio can attain a level of 12 dB. Its zero static power consumption, electrical programming (CMOS compatibility), low loss, high durability, and support for multi-level storage make it an ideal choice for building large-scale photonic neural network weight libraries. This approach has been demonstrated to effectively address the optoelectronic conversion bottleneck, facilitate the integration of optoelectronic co-packaging, and provide a scalable on-chip, non-volatile storage solution for energy-efficient neuromorphic optical engines.

**Figure 9:**
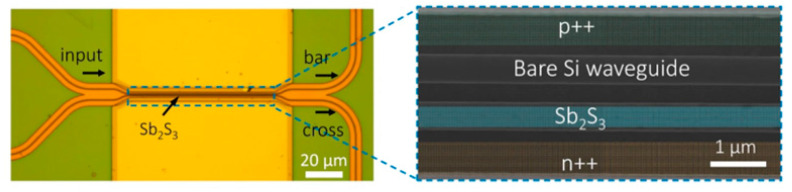
Optical and SEM images of asymmetric directional couplers of Sb_2_S_3_-Si hybrid waveguides [181].

As is widely documented in the extant research, conventional PCM photonic devices typically face three substantial challenges: high optical absorption, which limits phase-modulation applications; insufficient electrically controlled cycle life; and multi-level operation, which relies on complex optical and electrical tuning. The electrically programmable multi-bit integrated photonic platform based on the wide-bandgap phase-change material Sb_2_S_3_, as illustrated in Figure 9, addresses the fundamental limitations of conventional programmable photonic integrated circuits for static power consumption, loss, and multi-level operation [181]. This work achieves ultra-low loss and high extinction ratio through an innovative silicon PIN diode microheater structure and low-temperature atomic layer deposition aluminum oxide packaging technology. The work demonstrates a <1.0 dB insertion loss and >10 dB extinction ratio in the communication O band, which is significantly better than the high absorption defects of traditional GST-based devices. Concurrently, unprecedented advancements in durability have been realized, showcasing the electrically controlled reversible switching of Sb_2_S_3_ for over 1600 stable cycles in an asymmetric directional coupler. These findings lay the foundation for large-scale integration. Furthermore, the utilization of the cumulative effect of identical pulse sequences has enabled the precise control of 32 states in a directional coupler, with a resolution of 0.50 ± 0.16 dB per step. Additionally, dynamic pulse control has been employed to successfully correct π-level random phase errors in a Mach–Zehnder interferometer. These advances are rooted in the polycrystalline nature of Sb_2_S_3_, which undergoes a pulse number-dependent phase transition, and in an optimized thermal management design that achieves a programming energy density of approximately 10 fJ/μm^3^. The device exhibits zero static power consumption, CMOS-compatible electrical programming, and nanoscale packaging, which collectively offer a highly efficient solution for weight storage and dynamic reconfiguration in photonic neuromorphic engines. This solution is particularly well-suited for CPO applications requiring low-frequency programming, such as optical computing, quantum information processing, and post-fabrication calibration.

**Figure 10:**
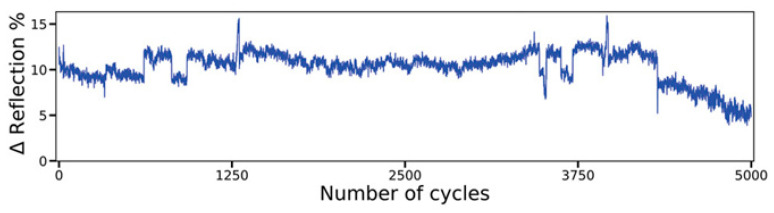
Changes in reflection between the two phases of Sb_2_Se_3_ during 5000 phase-change cycles [182].

The innovative application of chalcogenides, specifically Sb_2_S_3_ and Sb_2_Se_3_, as ultra-low-loss reversible phase-change materials in photonic integrated circuits, has effectively addressed the primary challenge posed by high optical absorption loss of conventional phase-change materials (such as GST) [182]. The 40 nm film prepared by magnetron sputtering exhibits almost zero intrinsic absorption (k < 10^−5^) in the 1550 nm communication band, while achieving significant refractive index modulation. The moderate absorption characteristics of Sb_2_Se_3_ in the visible light band (638 nm) support conventional laser programming, and experiments have verified >4000 stable reversible phase-change cycles (better than 1000 times for Sb_2_S_3_), overcoming the material degradation problem caused by sulfur/selenium volatilization in traditional PCM, as shown in Figure 10. When integrated into a silicon waveguide, 25 nm thick Sb_2_Se_3_ in the crystalline state exhibits a propagation loss of merely 0.01 dB/μm, which is two orders of magnitude lower than that of GST. Sb_2_Se_3_ demonstrates a remarkable enhancement in its performance, achieving 29 rad/dB. This represents a substantial improvement of over tenfold compared to the standard of GST, which measures at 0.282 rad/dB. Notably, this enhancement signifies the first instance of achieving a high degree of compatibility with silicon photonic modes, characterized by a field overlap exceeding 97%. These properties effectively address the three major challenges of high loss, limiting phase-control functionality, insufficient material durability, and mode mismatch, providing an ideal material platform for zero-static-power neuromorphic optical engine weight units and programmable phased arrays.

**Figure 11:**
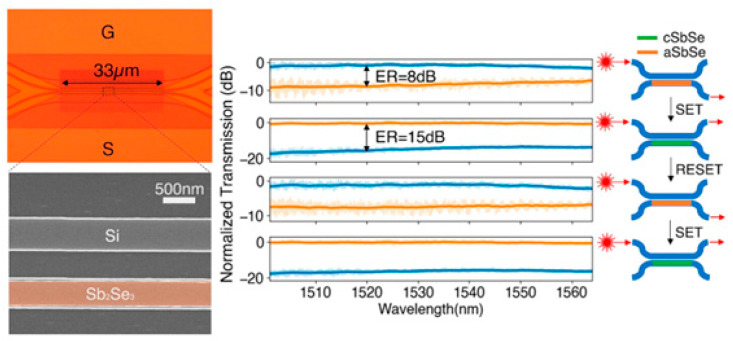
Electrical control of directional coupler switches [183].

The challenge of independently modulating the resonator coupling strength and internal phase due to thermal crosstalk, in conjunction with the persistent static power consumption necessary to preserve the system’s state, has necessitated the development of an innovative technology capable of arbitrarily controlling the racetrack-shaped resonator. This pivotal component of programmable photonic integrated circuits (PICs) has been achieved by employing low-loss phase-change materials (Sb_2_Se_3_) [183]. The directional coupler switch, which is non-volatile and ultra-compact (with a length of 33 μm), is based on Sb_2_Se_3_. Its performance is excellent, with extremely low insertion loss (approximately 0.36 dB at 1530 nm), a high extinction ratio, and excellent durability, as shown in Figure 11. The integration of this switch as a core unit within the racetrack-shaped resonator has enabled the realization of independent, non-volatile electrical programming control of the resonator coupling coefficient and internal phase, marking a pioneering advancement in the field. This breakthrough capability enables the arbitrary adjustment of the extinction ratio (ER) of the resonant peak without disturbing the resonant wavelength. The successful demonstration of precise control of five different extinction ratio levels, ranging from 0 dB to 34 dB, at a fixed wavelength, is a significant achievement. This achievement addresses the critical need for precise and energy-efficient control of resonator performance in applications such as optical interconnects and neuromorphic optical computing. The device’s features, including its ability to consume zero static power, its programmability that does not require constant maintenance, its compact size, its low-loss characteristics, and its reliability, are promising for the development of large-scale programmable photonic integrated circuits with zero static power consumption. These characteristics are particularly relevant for future high-performance optical engines.

A silicon photonics platform with ultra-low energy consumption, non-volatile programmability, and a foundation in graphene heaters and PCM offers essential technical support for high-efficiency optical engines and artificial neuromorphic optical computing [184]. The innovative use of single-layer graphene as an efficient and transparent microheater, combined with two phase-change materials, achieved breakthrough performance. Specifically, the GST-based 4.73 μm ultra-compact broadband optical switch achieved a switching contrast of 3 dB in the entire communication C-band under CMOS-compatible driving conditions. The current of 78 mA was observed, and the programming energy density was found to be as low as 8.7 ± 1.4 aJ nm^−3^. This value is more than 20 times lower than the most advanced technology at the time and approaches the theoretical limit. Additionally, the Sb_2_Se_3_-based microring phase shifter exhibited an insertion loss of only 0. The device demonstrated a noise level of 33 dB (6 μm long), exhibited 14 distinguishable non-volatile phase states, and showed a phase modulation efficiency of 0.496 V cm, surpassing the efficiency of conventional silicon-based phase shifters. Of particular significance is the observation that both devices demonstrated exceptional durability, with a switching cycle count exceeding 1000, and attained quasi-continuous multi-level modulation through the precise modulation of programming energy. These findings lay the groundwork for the development of high-efficiency, high-density “set and forget” photonic neuromorphic weight units and optical interconnect cores. This platform has been demonstrated to reduce dynamic energy consumption associated with photonic reconfigurability, and it has been shown to have zero static power consumption, CMOS-compatible drive, and compatibility with various media platforms. These characteristics position it as an ideal candidate technology for promoting the development of next-generation CPO optical engines and optical neural networks.

**Figure 12:**
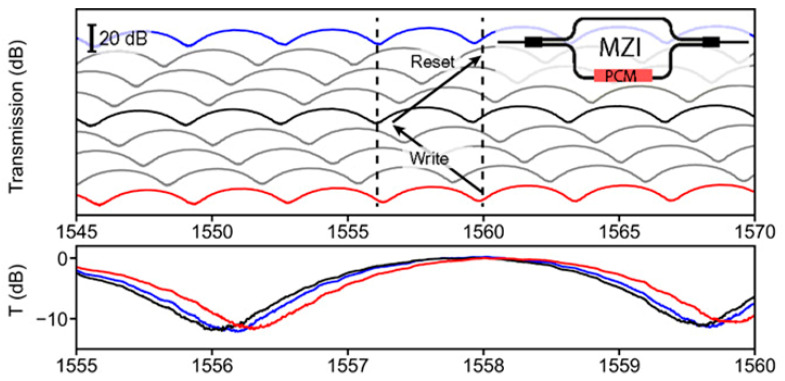
Tuning of MZI by switching ultra-low loss PCM to achieve selective optical phase control [58].

The ultra-low-loss phase-change material Sb_2_Se_3_ has achieved a breakthrough in non-volatile programmable silicon photonics, providing key technical support for high-density, low-power optical engines and neuromorphic photonic processors [58]. This research successfully overcame the core problem of traditional phase-change materials in the communication band, which exhibited high optical loss, making it difficult to decouple optical phase control from amplitude changes, which seriously limits the application of pure phase modulation. The researchers leveraged the ultra-low intrinsic optical loss (k < 10^−5^, approaching transparency in the communication band) and substantial refractive index contrast exhibited by Sb_2_Se_3_ in both amorphous and crystalline states, and for the first time, excellent, almost lossless optical phase control was achieved on a silicon photonics platform. In an MZI phase shifter based on Sb_2_Se_3_, a substantial reversible phase shift of more than 10π radians was successfully demonstrated. Concurrently, the insertion loss remained virtually constant throughout the phase-tuning process (no significant change in modulation depth), a feat heretofore unattainable with conventional phase-change materials, as illustrated in Figure 12. A particularly noteworthy aspect of this research is the introduction of the innovative concept of “nanophotonic digital patterning.” The direct laser writing of amorphous Sb_2_Se_3_ pixel patterns (with an 800 nm pixel pitch) onto a 33 μm × 6 μm multimode interferometer (MMI) has been demonstrated to achieve programmable routing control of the optical path. This has been evidenced by the demonstration of a 92%:8% splitting ratio. The device’s dimensions are significantly more compact than existing cascaded interferometer grid solutions. This technology exhibits excellent reconfigurability, with patterns capable of repeated writing and erasure, and low insertion loss, with an additional loss due to patterning of approximately 0.5 dB. While contemporary optical writing methodologies continue to require enhancement about durability—evidenced by contrast degradation following approximately 600 switching cycles and velocity—the present study addresses the fundamental impediment of strong coupling between phase and loss in phase-change photonics. This development signifies a novel technological trajectory for the prospective implementation of high-density, non-volatile, and low-loss programmable photonics.

**Figure 13:**
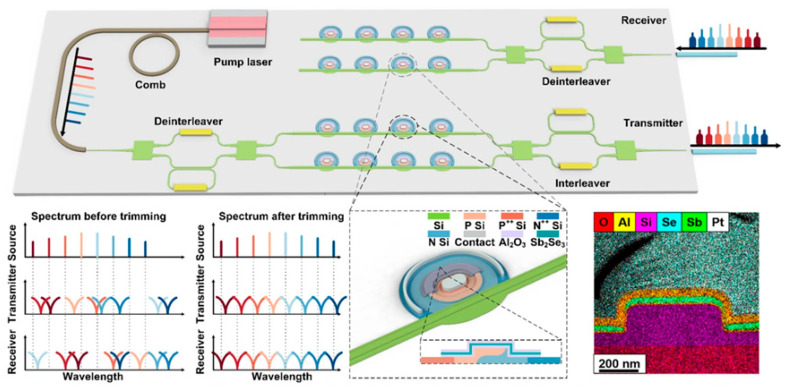
Application of PCM in artificial neuromorphic devices: schematic diagram of PCM-powered MRR transceiver structure and MRR optical engine structure with integrated PCM [185].

The programmable wavelength division multiplexing (WDM) optical transceiver technology, which is based on the monolithic integration of low-loss phase-change material Sb_2_Se_3_ (SbSe) and silicon-based PN junction MRR, has significantly promoted the development of high-density, low-power CPO light engines [185], as shown in Figure 13. The research successfully overcame the core bottlenecks of the practical application of silicon-based MRR. First, the resonant wavelength detuning caused by manufacturing deviations required post-calibration. Second, the high static power consumption and thermal crosstalk problems of traditional thermal tuning schemes were addressed. In an innovative approach, Sb_2_Se_3_ was deposited directly on the PN junction of the MRR. The Joule heat of the PN junction under a forward bias electric pulse is utilized to trigger the amorphizatio or crystallization of Sb_2_Se_3_. This approach enables non-volatile, field-programmable resonant wavelength fine-tuning. The resonant wavelength of a single MRR can be reversibly tuned over a range exceeding the free spectral range (FSR, 7.2 nm) with a minimum tuning resolution of ~10 pm. This objective is accomplished by modulating the electrical pulse width, which exhibits an ultra-low energy consumption of 0.4–5.8 μJ/pulse. The result is a tuning range that is both extensive and precise. Furthermore, after wavelength adjustment, the MRR’s modulation performance and detection performance remain virtually unaffected, preserving its core functionality thanks to the Al_2_O_3_ insulating layer protecting the PN junction. Additionally, a four-channel cascaded MRR array facilitates high-density system integration. Sb_2_Se_3_ trimming has been demonstrated to evenly align the initially squeezed resonance peaks within the free spectral range (FSR), thereby enabling parallel modulation and detection at a rate of 4 × 100 Gbps. Fourthly, the subject of zero-static power thermal stability is addressed. The innovative use of a single MRR as an on-chip optical power monitor, in conjunction with thermoelectric cooler feedback control, maintains a 25 Gbps signal error rate stable at 10^−7^ under ambient temperature fluctuations of ±2.3°C, thereby eliminating the static power consumption of traditional on-chip heaters. This solution utilizes the MRR’s original PN junction for phase-change triggering, thereby eliminating the requirement for additional heating electrodes. This approach is designed to optimize chip area savings while preserving the MRR’s compact dimensions. This addresses the limitations of traditional post-trim technology, including limited scope and complex processes, and provides a key technology path for wafer-level integration of next-generation high-density, energy-efficient CPO optical engines.

The integration of PCMs within silicon-based photonic integrated circuits represents a pivotal approach to the realization of non-volatile, low-static-power optoelectronic devices. However, this integration process faces a fundamental challenge: the thermal stability of mainstream chalcogenide PCMs is typically lower than that required during high-temperature steps (such as source-drain annealing) in standard CMOS front-end-of-line (FEOL) processes. This phenomenon results in uncontrolled crystallization, element interdiffusion, and performance degradation, thereby hindering their direct application in front-end or mid-level processes. To address this challenge, the research community has devised two advanced, fab-compatible back-end-of-line integration strategies that effectively incorporate PCMs into functional systems without compromising the performance of the original circuits.

The initial strategy entails monolithic back-end-of-the-line (BEOL) integration, underpinned by an etch stop layer [186]. In this approach, a SiN etch stop layer is deposited on the planned functional area waveguide in advance during the standard silicon photonics wafer manufacturing process. Subsequent to the completion of conventional CMOS interconnects and passivation layer deposition, a deep trench is etched in the cover layer using an etch process with high selectivity for SiO_2_/SiN. This process stops at the SiN layer, thereby ensuring the protection of the underlying silicon waveguide structure. Subsequent to the removal of the SiN, a PCM film is deposited in the trench by means of a low-temperature process, such as sputtering. The primary benefit of this approach is that it does not necessitate any modifications to the process design kit (PDK), while concurrently introducing minimal additional losses. This offers a substantial and viable route for the fabrication of wafer-level, high-yield PCM devices on a commercial silicon photonics platform. The second strategy involves 3D heterogeneous integration, which is based on wafer bonding and substrate removal [187]. This solution utilizes advanced hybrid bonding technology to bond the processed PIC wafer and the electronic CMOS wafer to an intermediate adapter plate. It then completely removes the silicon substrate and buried oxide layer of the PIC wafer, thereby exposing the back side of the device layer silicon waveguide. This process forms a clean, topological defect-free new integration interface. PCMs can be deposited and patterned on this novel surface through low-temperature processes. Despite the solution’s elevated process complexity, it attains complete decoupling of new material integration from the original CMOS/BEOL process. This not only circumvents thermal budget conflicts but also affords enhanced design freedom. For instance, the metal layer in the original BEOL can be utilized as an efficient bottom reflector, thereby significantly enhancing the performance of passive devices such as optical couplers and antennas. Despite the inherent thermal incompatibility between PCMs and CMOS FEOL processes, the aforementioned back-end integration solutions have emerged as reliable technical pathways for this field, offering clear solutions compatible with advanced manufacturing platforms.

It has been demonstrated that, by leveraging PCM, the silicon photonic platform can support non-volatile, multi-level optical modulation functions. This finding underscores the considerable promise of PCM in the reconstruction of photonic devices. It is noteworthy that, in addition to PCM, ferroelectric materials have also garnered considerable attention in recent years as a promising class of non-volatile photonic materials. These devices have demonstrated distinct advantages, including pure phase modulation, low loss, and low power consumption [188]. The direction of the polarization of the ferroelectric domain in the BTO film can be regulated by an electric field, thereby altering the effective Pockels coefficient. This, in turn, enables the realization of an optical phase shift based purely on the real part of the refractive index modulation. In contrast to the PCM mechanism, which is based on the crystalline-amorphous phase transition, BTO can achieve phase shift with minimal light absorption due to its ferroelectric domain flipping mechanism. Its unit device insertion loss can be as low as 0.07 dB, which is significantly lower than that of typical PCM modulators. Furthermore, the switching energy of the BTO device is notably reduced, with a minimum of only 4.6 pJ required to achieve state switching. In contrast, the crystallization process of PCM frequently necessitates energy levels of the order of nJ, particularly in repeated operations, where the discrepancy in energy consumption is more pronounced. With regard to multi-bit storage capacity, BTO has attained a stable electrical programming state of more than 3 bits and has demonstrated favorable state retention characteristics, which are commensurate with the performance of advanced PCM materials such as GSST and Sb2Se3. The relevant progress is shown in Table 6.

Notwithstanding this observation, substantial disparities persist between the two material systems with respect to their technological development stages and integration characteristics. PCM’s most significant advantage lies in its extremely compact device size, which facilitates high-density integration. Conversely, BTO necessitates millimeter-scale π phase shifts to attain this objective, a substantial constraint in numerous high-integration applications. Additionally, PCM’s compatibility with silicon-based photonic back-end processes has been thoroughly demonstrated, bolstered by the maturity of the fabrication processes and the reliability data available. However, issues such as ferroelectric materials’ large-scale compatibility with CMOS processes, consistent film quality, and long-term domain stability require further research. From an application perspective, the two also exhibit a degree of complementarity. PCM, with its high extinction ratio and subwavelength dimensions, is particularly well-suited for optical switches, intensity modulators, and integrated storage and computing photonic networks. Ferroelectric materials such as BTO possess unique advantages in low-coherence-noise, low-power phased arrays, optical neural networks, and quantum optical information processing systems due to their absorption-free phase-modulation capabilities and picojoule-level operation energy consumption. The integration of PCM with ferroelectric materials has been shown to enhance the technological repertoire available for non-volatile photonic devices, offering a range of solutions that can be tailored to meet the distinct requirements of various systems and integration scales. The proposed back-end integration strategy is not only applicable to the heterogeneous integration of PCM and silicon photonics platforms but also provides a potential technical path for the future introduction of ferroelectric materials and other functional material systems. Future research endeavors may concentrate on integrating these two categories of materials to achieve multifunctional coordination, regulated integration, and three-dimensional hybrid integration, thereby further propelling the advancement of high-performance, programmable photonic processing chips.

## 5. Conclusions

The accelerated growth of AI computing clusters, the ongoing expansion of data centers, and the impending advancements in 6 G networks are collectively propelling a profound and pervasive digital transformation on a global scale. This transformation is generating an exponential surge in demand for ultra-high communication bandwidth, minimal transmission latency, and optimized energy efficiency. This article systematically examines and analyzes the key advancements and developments in CPO technology. At the level of package integration architecture, the present article focuses on various CPO implementations based on advanced packaging technologies, including TSV-based 2.5D/3D integration solutions and the emerging field of glass interposer technology utilizing TGV. The text goes on to analyze the potential for building high-density, low-crosstalk optoelectronic interconnect platforms, as well as the potential and application challenges of FOWLP in CPO optical engine integration. The present article is an examination of the key waveguide fabrication processes on glass substrates as they relate to core optical interconnect technology. A thorough comparison and evaluation of two highly competitive approaches was conducted. Femtosecond laser direct writing waveguides boast three-dimensional processing capabilities, high design freedom, and excellent compatibility with glass substrates, offering unique advantages for fabricating waveguides with complex topologies. Ion exchange waveguide technology has emerged as a significant avenue for the fabrication of cost-effective and highly consistent optical interconnects. This technological advancement, characterized by its mature process, affordability, and scalability, has become a prominent solution for achieving these objectives. The two processes, distinguished by their divergent principles, optical performance (loss, mode characteristics), manufacturing costs, and application scenarios, present a range of technical options for CPO optical interconnect design. The paper also analyzes the key mechanisms and optimization strategies for chip–waveguide–fiber coupling, their advantages in process tolerance and planar integration, and their respective applicable scenarios and technical bottlenecks that need to be overcome. Furthermore, the paper proactively explores the disruptive potential of PCM-based novel artificial neuromorphic photonic devices. PCM’s distinctive light-induced, non-volatile phase-change properties emulate the synaptic weight-control behavior of biological neurons, thereby establishing a physical basis for the direct implementation of integrated storage and computation photonic neural networks within CPO architectures. The integration of high-efficiency optical interconnects (CPO) with intelligent optical computing is anticipated to overcome the energy efficiency and processing speed limitations of conventional von Neumann architectures. This integration has the potential to create new opportunities for the development of future artificial intelligence (AI) hardware. This development signifies a strategic innovation in CPO technology, representing a transition from a focus on “connectivity” to one of “intelligence.” The integration of optoelectronics and optical technologies into CPO technology is a significant development that has the potential to overcome the limitations of data transmission in the post-Moore era. The joint efforts of continuous exploration and coordinated optimization of advanced packaging architectures, core optical interconnect processes, efficient coupling solutions, and emerging intelligent photonic devices will collectively propel CPO from the laboratory to large-scale commercial use. This will establish a robust technical foundation for addressing the pressing demands of AI, 6 G wireless networks, and next-generation data centers for achieving extreme bandwidth, ultra-low latency, and ultra-high energy efficiency. This development signifies the advent of a novel era of optoelectronic integration for high-performance computing and communication systems.

## Figures and Tables

**Table 1:** FOWLP technology progress.

Ref.	Transfer Rate (Gbps)	Modulation Mode	Bandwidth	Channel	Insertion Loss (dB)	Return Loss (dB)	Package Size (mm)
[64]	200	PAM4	800 G	4	<0.05 @ 28 GHz	>10 @ 100 GHz	8.3 × 13
[65]	56	NRZ	400 G	8	<0.46 @ 56 GHz	>20 @ 40 GHz	12 × 12
[66]	106	PAM4	1.6 T	16	-	-	-
[69]	224	PAM4	1.79 T	6	<0.5 @ 56 GHz	<−18 @ 56 GHz	9.5 × 13
[67]	-	-	-	-	<0.5 @ 60 GHz	>20 @ 60 GHz	11 × 11
[9]	100	-	51.2 T	64	-	-	-
[72]	-	-	-	-	<0.5 @ 40 GHz	-	-
[73]	800	PAM4	3.2 T	4	1.5 @ 26 GHz	-	-

**Table 2:** Progress of TSV/TGV-based packaging.

Ref.	Transfer Rate (Gbps)	Modulation Mode	Bandwidth	Channel	Insertion Loss (dB)	Packaging	Through-Hole	Bit Error Rate	Power (Pj/Bit)
[81]	11.3	NRZ	-	4	<1.1 @ 75 GHz	2.5D	TSV	10^−9^	-
[82]	64	PAM4	256 G	4	<−1.55 @ 75 GHz	2.5D	TSV	-	6
[84]	100	PAM4	3.2 T	32	2–3	2.5D	TSV	10^−8^	5
[87]	100	PAM4	400 G	4	3.5	3D	TSV	-	-
[88]	112	PAM4	-	-	0.35	3D	TSV	-	-
[89]	224	PAM4	102.4 T	-	0.72	2.5D	TGV	-	-
[90]	100	PAM4	400 G	4	−1.5	2.5D	TGV	10^−7^	-
[91]	25	NRZ	400 G	16	-	3D	TGV	10^−12^	-

**Table 3:** Progress in FLDW.

**Ref.**	[115]	[118]	[119]	[120]	[121]
**Waveguide size (um)**	1–50	4.9–26.5	8–18	≈10	13 × 16
**Refractive index contrast**	5.8 × 10^−4^	5 × 10^−4^	10^−5^	10^−2^	3.5 × 10^−3^
**Propagation loss (dB/cm)**	<0.2	0.31–0.34	0.2	0.23	0.28
**Coupling loss (dB)**	-	-	0.045	0.084	-
**Wavelength**	840/1550	450–1550	980–1610	1310/1550	1890
**Depth (** **μm** **)**	5–500	150	20–170	150	150
**Thermal stability**	250 °C	Room temperature	-	-	-
**Laser parameters**	5 kHz, 224 nJ	1 MHz, 20–30 nJ	1 MHz, 28–30 nJ	1087 kHz, 123 nJ	5 MHz, 90 nJ

**Table 4:** Progress of ion-exchange glass waveguides.

Ref.	Waveguide Type	Material	Propagation Loss (dB/cm)	Coupling Loss (dB)	Wavelength (nm)	Panel Size (mm)	Reliability
[129]	Single mode	PTR glass	0.08	0.47	1550	76.2 × 1	-
[130]	Single mode	Alkali glass	0.034	0.31	1310	150 × 150	1000 h (130 °C/85%RH)
[131]	Single mode	Alkali glass	0.08	0.3	1310	100 × 50	1000 Cycle (−40~125 °C)
[132]	Single mode	Molten glass	<0.1	0.5	1310	420 × 255	-
[133]	Single/multimode	D263T	~0.15	0.15	1550	303 × 227	1000 Cycle

**Table 5:** Progress of optical coupling.

Ref.	Platform	Coupling Method	Coupling Efficiency	Insertion Loss (dB)	Crosstalk (dB)	Bandwidth (nm)
[141]	SOI	Edge coupling	~62%	<2	<−10	90
[142]	SOI	Edge coupling	>87%	−0.6	<−25	@ 1550
[143]	SOI	Edge coupling	>98%	>−0.6	<−19	C-band
[148]	SiP PIC	Edge coupling	>85%	<1	-	-
[150]	Glass substrate	Flip-chip + VCBEL	-	1.6	-	O-band
[151]	LNOI	Edge coupling	-	1.16	-	@ 1550
[153]	SOI	Photonic wire bond	-	0.73 dB	-	@ 1550 nm
[154]	SiN	Free-form micro-optical reflector	-	0.5 dB	-	@ 1550 nm
[156]	SiN	Apodized grating coupling with aluminum back reflector	88%	-	-	C-band

**Table 6:** Progress of artificial neuromorphic devices based on PCM.

**Ref.**	[180]	[181]	[182]	[183]	[184]	[58]	[185]	[188]
**Device**	P-RAM memory	Directional coupling switch	Directional coupling switch	Microring switch	Phase modulator	MZI Phaser	MRR	Ferroelectric phase shifter
**Material structure**	Si-GSSe	Si-Sb_2_S_3_	Si-Sb_2_Se_3_	Si-GST	Si-Sb_2_Se_3_	Si-Sb_2_Se_3_	Si-Sb_2_Se_3_	Si/BaTiO_3_/Au
**Insertion loss (dB)**	0.12	<1.0	0.36	0.5	0.002	<0.02	0.6	0.07
**Extinction ratio (dB)**	12	10–15	15/8	3	-	-	13.6	-
**Energy (nJ)**	1.5	11.6–29.2	0.57	5.55	9.25	~10	5.8	4.6 × 10^−3^
**Durable cycle**	5 × 10^5^	>1600	>1000	>1500	>2000	>500	>100	>300
**Response time**	0.5 ms	200 ms	100 µs	100 µs	100 µs	50 ms	1 ns	100 μs
**Phase coordination**	-	-	-	-	0.0082π	>10	-	0.15π
**Wavelength** **coordination**	-	0.39	-	-	0.021	-	7.2	-
**Multi-level bit width**	4	5	5	7	14	-	-	>3
**Device length (um)**	80	79	33	4.7	6	250	13	150

## Data Availability

Not applicable.
